# Neutrophil activation and NETosis are the major drivers of thrombosis in heparin-induced thrombocytopenia

**DOI:** 10.1038/s41467-019-09160-7

**Published:** 2019-03-21

**Authors:** José Perdomo, Halina H. L. Leung, Zohra Ahmadi, Feng Yan, James J. H. Chong, Freda H. Passam, Beng H. Chong

**Affiliations:** 1 Haematology Research Unit, St George and Sutherland Clinical School, Faculty of Medicine, University of New South Wales, Sydney, NSW Australia; 2 Sydney Medical School, The University of Sydney, Sydney, NSW Australia; 3 Centre for Heart Research, Westmead Institute for Medical Research, The University of Sydney, Sydney, NSW Australia; 4 Department of Cardiology, Westmead Hospital, Sydney, NSW Australia; 5 Victor Chang Cardiac Research Institute, Darlinghurst, NSW Australia; 6 New South Wales Health Pathology, St George and Sutherland Hospitals, Sydney, NSW Australia

## Abstract

Heparin-induced thrombocytopenia/thrombosis (HIT) is a serious immune reaction to heparins, characterized by thrombocytopenia and often severe thrombosis with high morbidity and mortality. HIT is mediated by IgG antibodies against heparin/platelet factor 4 antigenic complexes. These complexes are thought to activate platelets leading to thrombocytopenia and thrombosis. Here we show that HIT immune complexes induce NETosis via interaction with FcγRIIa on neutrophils and through neutrophil-platelet association. HIT immune complexes induce formation of thrombi containing neutrophils, extracellular DNA, citrullinated histone H3 and platelets in a microfluidics system and in vivo, while neutrophil depletion abolishes thrombus formation. Absence of PAD4 or PAD4 inhibition with GSK484 abrogates thrombus formation but not thrombocytopenia, suggesting they are induced by separate mechanisms. NETs markers and neutrophils undergoing NETosis are present in HIT patients. Our findings demonstrating the involvement of NETosis in thrombosis will modify the current concept of HIT pathogenesis and may lead to new therapeutic strategies.

## Abstract (web summary)

The pathogenesis of heparin-induced thrombocytopenia and thrombosis (HIT) is mediated by heparin-reactive autoantibodies binding to platelets (thrombocytes). Here the authors show neutrophil activation and NETosis are elevated in patients with HIT, and are essential for thrombosis in HIT mouse models.

## Introduction

Adverse drug effects are common in clinical practice and often have negative impact on patient safety. Among these, adverse effects caused by anticoagulants are concerning to clinicians, particularly those caused by heparin, a widely used anticoagulant. Heparin and heparin-derived drugs (including unfractionated heparin, low-molecular-weight heparin and occasionally fondaparinux) may induce an immune reaction, termed heparin-induced thrombocytopenia (HIT). HIT is a hypercoagulable state, which often causes severe and extensive thrombosis (both venous and arterial) that results in high morbidity and mortality^1^. The thrombotic complications include severe limb thrombosis and gangrene requiring limb amputation, life-threatening pulmonary embolism, acute myocardial infarction and stroke^2^, and also characteristic thrombosis at distinctive sites (bilateral adrenal infarct, portal and intestinal vein and cerebral sinus thrombosis). It is ironic that patients with HIT develop severe thrombosis when they are also thrombocytopenic and are receiving heparin, a potent anticoagulant.

As an immune drug reaction, HIT occurs more frequently than other drug-induced immune thrombocytopenias;^1–4^ HIT occurs in about 3% of medical patients and about 5% of surgical patients receiving heparin. Furthermore, thrombosis is observed in as many as 50% of untreated HIT patients^3,5^. Data from clinical trials show that despite treatment with non-heparin potent anticoagulants (argatroban and lepirudin)^6^, the devastating clinical outcomes of HIT patients with thrombosis remain unacceptably high (Argatroban-915^7^ and HAT-1, 2 and 3^8^ studies). The reported incidence of thrombotic gangrene requiring limb amputation ranges from 5.5 to 14.8% and the mortality rate 11.9 to 23.1%^7,8^.

Consequently, there is an urgent clinical need to fully understand the pathogenesis of HIT. In particular, understanding the mechanism(s) of its thrombotic complications will improve management of this limb- and life-threatening condition and allow novel drugs to be developed for its more efficacious treatment. The current concept of the pathogenesis of HIT is that it is mediated by IgG autoantibodies that recognise complexes formed by platelet factor 4 (PF4) and heparin. The heparin/PF4/antibody immune complex, termed HIT immune complex (HIT IC) in this paper, engages FcγRIIa on the platelet surface, which leads to platelet activation, release of procoagulant factors, microparticles and platelet clearance^3^. According to current understanding, platelet activation is the main driver of the thrombotic process in HIT. Apart from platelets, other cell types such as monocytes contribute to the immunogenicity of the heparins. Monocytes and endothelial cell involvement has also been reported in the development of thrombosis in HIT^6,9^, but the roles of these cells in mechanisms of thrombosis in HIT are yet to be fully elucidated.

Recently, neutrophil extracellular traps (NETs) are increasingly being reported in patients with infection and thrombosis associated with various autoimmune and non-immune disorders^10–13^. NETs are DNA-containing structures released by neutrophils that incorporate intracellular factors, such as histones, myeloperoxidase (MPO) and elastase. NETs have a central role in infection, host defence and thrombosis^14–16^. NETs promote thrombin generation^17^ and, in turn, activated platelets promote NETs formation^18,19^, which are proposed to occur most likely through neutrophil–platelet interaction mediated by P-selectin^20^. NETs contain prothrombotic molecules, such as tissue factor, protein disulphide isomerase, factor XII^21^, von Willebrand Factor (VWF) and fibrinogen^22^. However, the presence of NETs or their contribution to hypercoagulability in HIT remains unexplored.

Here, we provide substantial evidence that NETosis occurs in HIT. More importantly our data show that HIT ICs can directly activate neutrophils, inducing these cells to undergo NETosis, without needing interaction with activated platelets. Furthermore, neutrophil activation and the resulting NETosis are sufficient and essential for development of thrombosis in HIT, suggesting that neutrophil activation is the major driver of thrombosis in HIT. This is contrary to the present prevailing concept that platelet activation is the key driving force of the thrombotic process in HIT. Our findings will contribute to the further understanding of HIT pathogenesis, may change its current concept and lead to new treatments that could improve the clinical outcomes of patients with HIT and thrombosis.

## Results

### Increased levels of NETosis markers in HIT patients

**Fig. 1:**
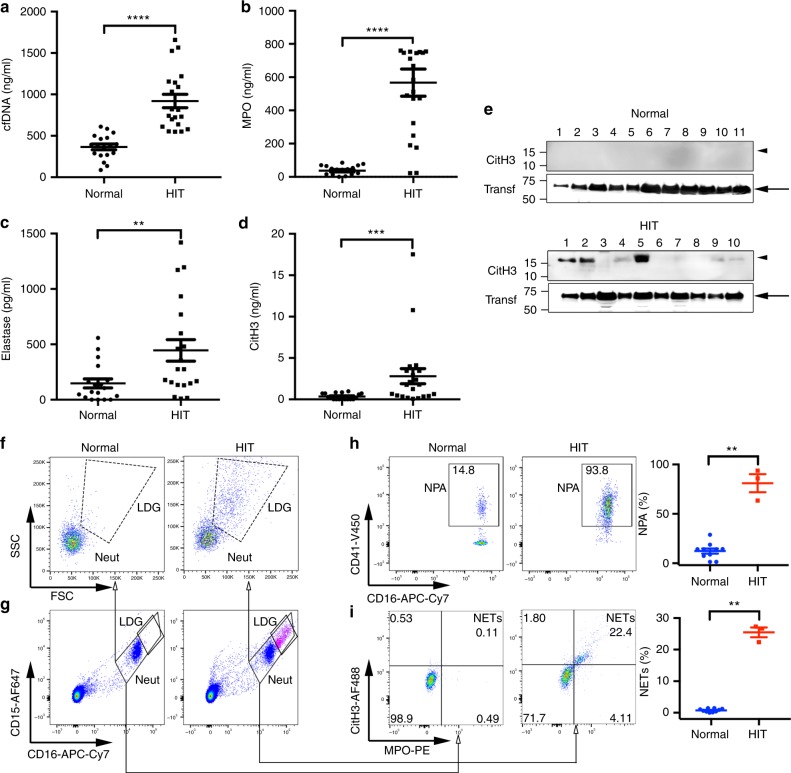
NETs are present in HIT patients. **a** cfDNA in HIT patients’ plasma (*n* = 21) relative to normal controls (*n* = 18) was detected using PicoGreen dsDNA fluorescence assay. **b** MPO levels in HIT patients’ plasma (*n* = 21) and normal controls (*n *= 18) were measured by ELISA. **c** Neutrophil elastase concentration in patients’ plasma (*n* = 20) relative to normal controls (*n* = 18) and **d** CitH3 levels in HIT patients’ plasma (*n* = 21) relative to normal controls (*n* = 18) was determined by ELISA. **e** Western blot images of CitH3 probed with anti-CitH3 antibody in normal controls and HIT patients’ plasma. Each lane represents a different donor’s plasma. Transferrin (Transf) was used as a loading control. Arrowhead indicates CitH3 band. Arrow denotes transferrin. **f** Representative flow cytometry density plots using fresh blood backgated for neutrophils (CD15^+^ CD16^+^ population shown in **g**). **g** Flow cytometric determination of neutrophils (CD15^+^ CD16^+^ population, Neut.). LDGs within the Neut population are shown. **h** Neutrophil–platelet aggregates (CD41^+^ events within the CD15^+^ CD16^+^ population). The graph shows the quantification of the flow cytometry data shown on the left in healthy controls (*n* = 10) and HIT patients (*n* = 3). NPA, neutrophil–platelet aggregates. **i** Representative dotplot of NETs present in vivo in healthy controls (left panels) and HIT patients (middle panel). The numbers in the quadrants indicate percentage of gated events. NETs were defined as CitH3 and MPO double positive events within the CD15^+^ CD16^+^ population. The graph shows the quantification of the flow cytometry data shown on the left in healthy controls (*n* = 10) and HIT patients (*n* = 3). Statistics, Mann–Whitney test. *P < 0.05; **P < 0.01; ****P < 0.0001. Mean ± s.e.m. shown in all graphs. LDG, low-density granulocytes, Neut, neutrophils. Source data for (**a**, **b**, **c**, **d**, **e**, **h**, **i**) are provided as a Source Data file

To determine whether NETs are generated in HIT, we examined plasma from HIT patients for the presence of NETosis markers: cell-free DNA (cfDNA), MPO, elastase and citrullinated histone H3 (CitH3) relative to normal controls. Significantly increased levels of cfDNA, MPO, elastase and CitH3 were detected in HIT patients’ plasma relative to controls (Fig. 1a–d). Thrombosis (including venous thromboembolism (VTE) and arterial thrombosis) was present in 18 out of 21 HIT patients. Three patients who received heparin as VTE prophylaxis developed thrombocytopenia, but no clinically overt thrombosis (isolated HIT). However, these patients were not screened by imaging investigations for the presence of subclinical thrombi. Plasma levels of VWF were also significantly higher in HIT patients relative to controls (Supplementary Fig. 1a). By western blotting, CitH3 was observed in the plasma of seven HIT patients (*n* = 10), while no CitH3 was observed in normal sera (*n* = 11) (Fig. 1e).

Next, we examined whether NETs were present in patients with active HIT. Fresh whole blood from HIT patients with acute HIT or normal controls was analysed by flow cytometry^23^ for the presence of NETs (for gating strategy see Supplementary Fig. 1b). As shown in Fig. 1f, neutrophils (backgated from CD15^+^ CD16^+^ population (Neut) in Fig. 1g, and shown as a side and forward scatter plot) from HIT patients included a second population of high-scatter cells, which were not present in healthy controls (Fig. 1f, g, LDG). These enlarged cells have been described as activated neutrophils^24^ or low-density granulocytes (LDGs)^25^ and have been observed in pathological conditions. We confirmed the presence of LDGs in fresh blood treated with HIT IgG and heparin (Supplementary Fig. 1c). Moreover, a large proportion of neutrophils were incorporated in neutrophil–platelet aggregates in HIT patients relative to controls (CD16^+^ CD41^+^ events) (Fig. 1h). NETting neutrophils, defined as MPO^+^ CitH3^+^ events within the CD15^+^ CD16^+^ population were also abundant in HIT samples, while signs of NETosis were undetectable in healthy controls (Fig. 1i). Fluorescence minus one controls are shown in Supplementary Fig. 1d. These findings suggest that NETs may be involved in the pathogenesis of HIT.

### HIT IgG induces NETosis and NETs-containing thrombi

**Fig. 2:**
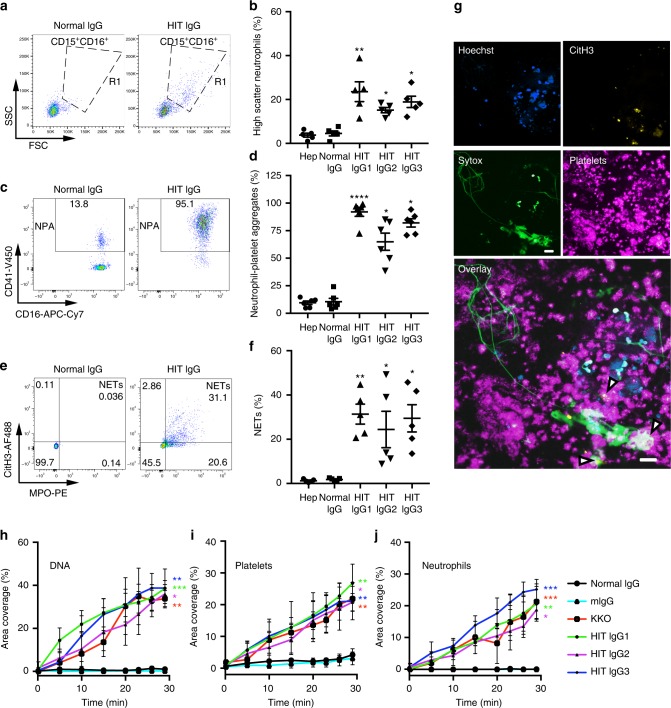
HIT patients’ IgG induces NETosis. **a** Representative flow cytometry density plots of side scatter (SSC) versus forward scatter (FSC) backgated for neutrophils (CD15^+^ CD16^+^ population). **b** Quantification of high-scatter neutrophils using IgG from three HIT patients repeated with blood from different healthy donors (*n* = 5). **c** Neutrophil–platelet aggregates (NPA) induced in whole blood by HIT IgG in the presence of heparin (0.5 U/ml), and (**d**) quantification of NPA using IgG from three HIT patients repeated with blood from different healthy donors (*n* = 6). **e** Representative flow cytometry scatter plots of NETs induced in whole blood by HIT IgG plus heparin. The numbers in the quadrants indicate percentage of gated events. **f** Quantification of NETs using IgG from three HIT patients with blood from different healthy donors (*n* = 5). **g** Citrullinated histone H3 is present in HIT IgG-induced thrombi ex vivo. DNA in nucleated cells was stained with Hoechst 33342 (blue), extracellular DNA with Sytox green (green), CitH3 with anti-CitH3 Alexa 594 (yellow) and platelets with anti-CD41 Alexa 647 (magenta). White areas indicate superposition of signals. White arrowheads indicate areas of CitH3 staining. Images were taken with a confocal laser-scanning microscope (Leica TCS SP8) with a ×40 water immersion objective running Leica’s LAS X software. Scale bar: 20 μm. **h–j** Graphs represent percentage area coverage of VWF-coated surfaces versus time for (**h**) the extracellular DNA (Sytox^+^ areas), (**i**) platelets (CD41^+^) and (**j**) neutrophils (CD15^+^). Percentage coverage area determinations were calculated for the times indicated. Mean ± s.d are shown; *n* = 3. Statistical analyses, for (**b**, **d**, **f**) Kruskal–Wallis test followed by Holm’s Stepdown Bonferroni procedure for adjusted *p*-values. Data are expressed as mean ± s.e.m. For (**h**, **i**, **j**), one-way ANOVA relative to normal IgG with Dunnett’s post-test. *P < 0.05; **P < 0.01; ***P < 0.001 relative to normal IgG. Mean ± s.d. are shown. Source data for (**b**, **d**, **f**, **h**, **i**, **j**) are provided as a Source Data file

Following detection of NETosis markers in HIT plasma and the presence of NETosis in HIT patients, we sought to determine whether plasma from HIT patients plus heparin was capable of inducing DNA release by purified neutrophils. All patient samples tested (*n* = 21) induced DNA release relative to normal controls (*n* = 18) (Supplementary Fig. 1e). This prompted us to examine the effect of purified HIT IgG plus heparin on whole blood. We observed changes in neutrophils (Fig. 2a, gate R1 and Fig. 2b) similar to those found in fresh blood of patients with active HIT (Fig. 1f). There was strong induction of neutrophil–platelet aggregates within an hour of treatment with HIT IgG and heparin (0.5 U/ml), but not in blood treated with heparin alone or normal IgG plus heparin (Fig. 2c, d). A large portion of neutrophils in HIT IgG-treated blood were also MPO^+^ CitH3^+^, which is indicative of NETs induction (Fig. 2e, f). Both neutrophil–platelet aggregates and NETs occurred only at therapeutic doses of heparin (0.5 U/ml) and were completely inhibited by supra therapeutic heparin concentration (100 U/ml) (Supplementary Fig. 1f and Fig. 1g), indicating that induction of NETs is dependent on the formation of heparin/PF4/HIT IgG complexes, where high heparin concentration disperses the complexes^26^. Therefore, inhibition of neutrophil–platelet complexes and NETs formation (MPO^+^ CitH3^+^ cells) in the presence of high heparin concentrations is most likely due to the absence of HIT immune complexes. Although heparin can destabilise already formed NETs by removing histones from the secreted DNA^22^, this is not observed at low-dose heparin (0.5 U/ml). High heparin concentration (100 U/ml) can disrupt HIT antibody–PF4–heparin complexes, but does not degrade NETs^27^. To investigate if NETs were generated during thrombus formation, we reconstituted the HIT condition in a microfluidics system using microchannels coated with VWF. Fresh whole blood was incubated with purified HIT IgG plus heparin at 37 °C and perfused into the microchannels at arteriole shear stress (67 dyne/cm^2^)^28^. The HIT condition reconstituted in this system led to significant thrombus formation. As illustrated in time-lapse movies, the thrombi were rich in platelets (Supplementary movie 1) and neutrophils (Supplementary movie 2) and produced substantial amounts of the extracellular DNA (Supplementary movie 3). Fluorescent images captured during video acquisition show thrombi composed of nucleated cells, extracellular DNA, fibrin and platelets (Supplementary Fig. 2a). At the end of the experiment (30 min of blood flow), the remaining thrombi were fixed and examined by confocal microscopy. DNA networks (Sytox^+^), CitH3^+^, Hoechst^+^-nucleated cells and platelet aggregates were observed in the clots (Fig. 2g). Quantitative analysis of percentage area coverage versus time shows marked deposition of extracellular DNA (Fig. 2h), platelets (Fig. 2i) and neutrophils (Fig. 2j) on VWF-coated microchannels for HIT compared with control. Extracellular DNA was found in conjunction with extensive fibrin deposition within the thrombus (Supplementary Fig. 2b). Addition of DNase I resulted in rapid fading of the Sytox fluorescence, thus confirming the presence of extracellular DNA in the thrombus (Supplementary movie 4 and Supplementary Fig. 3a, b). Therefore, HIT IgG ICs promote the formation of neutrophil–platelet aggregates and NETosis in vitro and induce the formation of thrombi containing neutrophils, extracellular DNA and CitH3 under flow conditions.

### NETs formation is a feature of HIT in vivo

**Fig. 3:**
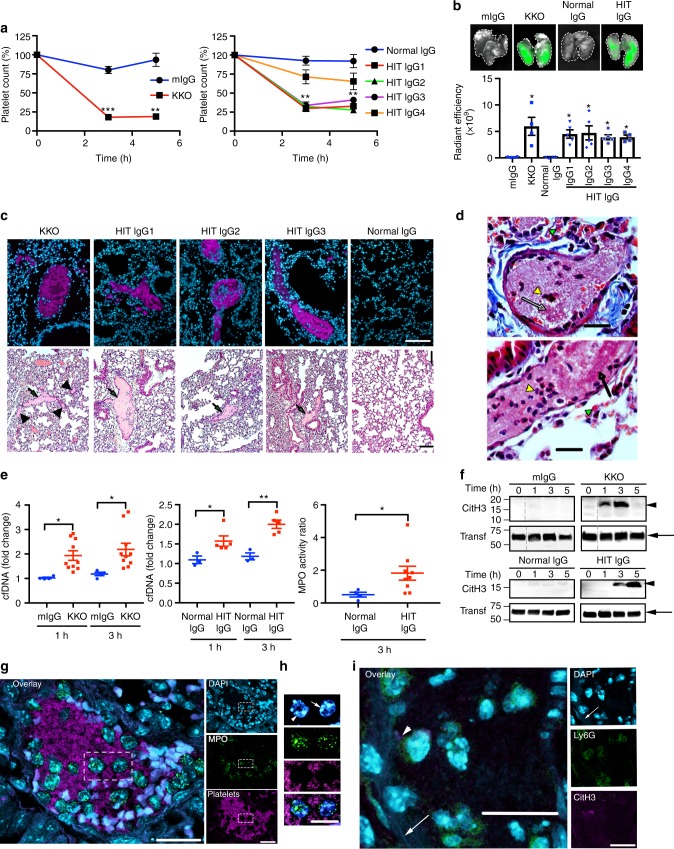
HIT IgG induces thrombosis and NETs in a HIT mouse model. **a** *FcγRIIa*^+^/*hPF4*^+^ mice were injected with KKO or control mouse IgG (*n* = 3, left panel) or HIT patients’ IgG or normal IgG (*n* = 4, right panel). Heparin administered at 1U/g. Platelet percentage was calculated relative to basal levels. **b** Images of mouse lungs harvested 5 h after treatment. Green fluorescence indicates anti-CD42c Dylight 649 platelet clots in lungs. Graph of lung fluorescence from mice treated in (**a**) (*n* = 4. For IgG1-3, *n* = 5). **c** Lung sections from mice treated as in (**b**). Upper panels show platelet-rich thrombi (magenta) which are present in all cases except with normal IgG. Blue, nuclei. Lower panels, H&E staining. Arrows indicate clots. Arrowheads show small clots in KKO-treated mice. 10X objective. Scale bar, 100 μm. **d** Carstair’s staining of KKO- (upper panel) or HIT IgG- (lower panel) induced thrombi in mouse lungs. Fibrin (orange–red, arrows); leucocytes (dark blue, yellow arrowheads); red blood cells (yellow–red, green arrowheads). Platelets (grey–blue) are mixed with fibrin. Scale bar: 20 μm. **e** cfDNA and MPO activity. Fold change in cfDNA in mouse plasma 1 h or 3 h after treatment with KKO (*n* = 10) or control mouse IgG (mIgG, *n* = 4) (left panel) or patient’s HIT IgG (*n* = 5) or normal control (*n* = 3, middle panel). Ratio of MPO activity at 3 h relative to time 0 following treatment with HIT IgG (*n* = 9) or normal control (*n* = 4, right panel). **f** Representative western blots of CitH3 in plasma from mice treated in (**a**). Arrowhead; CitH3. Arrow; transferrin (transf, loading control). Dotted lines: removal of irrelevant lanes. **g** Thrombi in mouse lung imaged by confocal microscopy. Platelets (magenta), nucleated cells (blue), MPO (green). Scale bar: 20 μm. **h** Magnified details of dotted area in **g** of nuclei (arrowhead) and decondensed neutrophil nucleus (arrow). Other panels denote MPO, platelets and overlay images, respectively. Scale bar: 10 μm. **i** Neutrophils were stained with Ly6G (green), CitH3 (magenta) and DNA (blue). Arrow, extracellular DNA; arrowhead, CitH3. Scale bar: 20 μm. Statistical analyses, (**a**, **e**) Mann–Whitney test. **b** Kruskal–Wallis test. *p*-values adjusted relative to normal IgG. Mean ± s.e.m. *P < 0.05; **P < 0.01; ***P < 0.001. Source data for (**a**, **b**, **e**, **f**) are provided as a Source Data file

The observations described above prompted us to explore the role of NETs in vivo in a mouse model of HIT. The HIT condition can be recapitulated using double transgenic mice expressing human platelet factor 4 (hPF4) and the R^131^ isoform of human FcγRIIa^29^ on their platelets to allow interaction with human IgG. FcγRIIa also interacts with mouse IgG1 and IgG2 immune complexes^30^. These mice (*FcγRIIA*^+^/*hPF4*^+^) also express FcγRIIa on other cells, including neutrophils (Supplementary Fig. 3c) and macrophages^31^. Development of HIT in these mice was evaluated by the extent of thrombocytopenia and, more specifically, by the degree of thrombus deposition in the lungs. *FcγRIIA*^+^/*hPF4*^+^ mice were treated with intravenous injections of the HIT-like monoclonal antibody KKO or IgG purified from HIT patients’ plasma followed by intraperitoneal administration of heparin (1 U/g). Mouse platelets were labelled in vivo with anti-CD42c Dylight 649 antibody. Administration of KKO or HIT IgG led to a significant decrease in platelet numbers (Fig. 3a) and a marked reduction in body temperature (Supplementary Fig. 3d) (other systemic reactions such as unresponsiveness to external stimuli were also observed together with hypothermia, features that have been described in patients with HIT). Examination of isolated, fixed lungs by micro CT scanning demonstrated significant accumulation of fluorescence (labelled platelets) in mice treated with KKO or HIT IgG plus heparin, indicative of pulmonary thromboembolism (Fig. 3b). Thrombi were not observed in animals treated with control mouse IgG or normal human IgG plus heparin (Fig. 3b). Examination of fixed lung sections by fluorescence microscopy and H&E staining confirmed the presence of thrombi lodged in pulmonary vessels of KKO- and HIT IgG-treated mice (Fig. 3c). These thrombi are fibrin rich and contain both scattered and clustered leucocytes (Fig. 3d and Supplementary Fig. 4). To determine if NETosis occurs during HIT, circulating markers of NETs were quantified in treated *FcγRIIA*^*+*^*/hPF4*^*+*^ mice. We observed significant increase in cfDNA and MPO activity in the plasma of mice treated with KKO or HIT IgG plus heparin (Fig. 3e). Assessment of plasma proteins by western blot showed the presence of CitH3 in KKO- or HIT IgG plus heparin-treated *FcγRIIA*^*+*^*/hPF4*^*+*^ mice but not in animals administered with control IgG plus heparin (Fig. 3f). Consistent with these observations, immunostaining of lung sections demonstrated evidence of NETosis in HIT IgG- and KKO-induced thrombi. MPO-containing cells were present in platelet-rich clots (Fig. 3g). Some neutrophils appeared non-activated, with distinctive lobulated nucleus (Fig. 3h, arrowhead), while others showed signs of nuclear decondensation, which is a characteristic of NETosis^32,33^ (Fig. 3h, arrow). Ly6G-positive cells (a surface marker for murine neutrophils) were the most abundant nucleated cells within the clots, and importantly both CitH3 and extracellular DNA were detected within clots (Fig. 3i). Collectively, these results show that NETosis is involved with HIT development in *FcγRIIA*^*+*^*/hPF4*^*+*^ mice and reiterate the observations in HIT patients and in ex vivo HIT reconstitution assays.

### Direct and indirect activation of neutrophils by HIT IgG

**Fig. 4:**
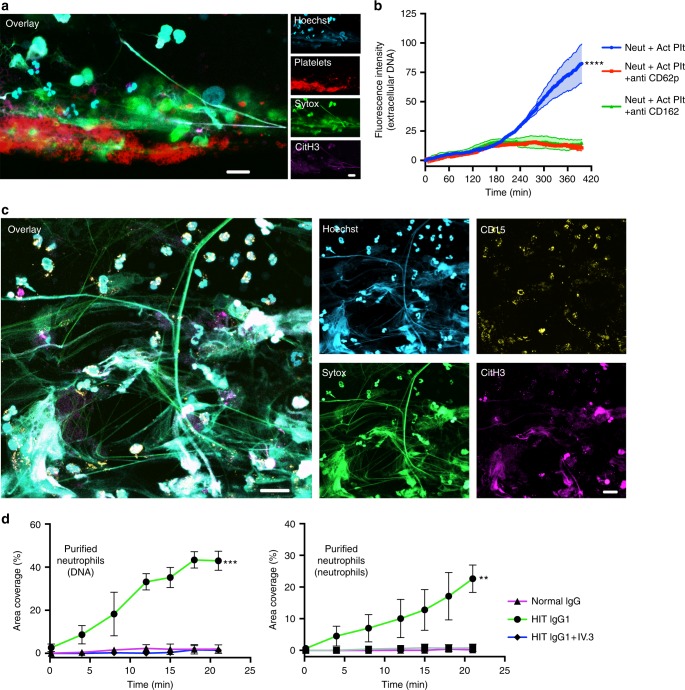
HIT IgG induces NETosis via activated platelets and by direct neutrophil activation. **a** Platelets and neutrophils were resuspended in autologous plasma at a 20:1 ratio and incubated with KKO plus heparin at 37 °C for 1 h, platelets were stained with anti-CD41 Alexa 647 (red), neutrophils with Hoechst (blue), extracellular DNA with Sytox green (green), and CitH3 with anti-CitH3 Alexa 594 (magenta) and then perfused on VWF-coated microchannels at a shear stress of 67 dynes/cm^2^ at 37 °C for up to 20 min. Samples were fixed at the end of the experiment. Panels show confocal images of a representative thrombus of three individual experiments, using different donors imaged with a 63x oil immersion objective. Scale bar: 20 μm. **b** Purified neutrophils were supplemented with HIT complex-activated platelets in the absence or presence of anti-CD62p or anti-CD162 and incubated at 37 °C. DNA release was monitored by confocal microscopy for over 5 h by Sytox green fluorescence. Total DNA, Hoechst staining. The graph shows the fluorescence intensity ratio of extracellular DNA/total DNA vs. time (*n* = 3). **c** Purified neutrophils were resuspended in autologous plasma and incubated with KKO plus heparin at 37 °C for 1.5 h. Neutrophils were stained with anti-CD15 Alexa 647 (yellow), DNA with Hoechst (blue), the extracellular DNA with Sytox green (green) and CitH3 with anti-CitH3 Alexa 594 (magenta) and perfused on P-selectin-coated microchannels at a shear stress of 67 dynes/cm^2^ at 37 °C for up to 30 min. Samples were fixed at the end of the experiment. The panels show confocal images of a representative thrombus of three individual experiments, using different donors imaged with a ×40 water immersion objective. Scale bar: 20μm. **d** Percentage area coverage versus time of P-selectin-coated surfaces perfused with purified neutrophils in the presence of HIT IgG, normal IgG or HIT IgG plus IV.3 antibody (all in the presence of heparin). DNA (left panel), neutrophils (right panel). *n* = 3; Mean ± s.d. is shown. Statistics: one-way ANOVA with Tukey’s correction for multiple comparisons. **P < 0.01; ***P < 0.001**;** ****P < 0.0001. Source data for (**b**, **d**) are provided as a Source Data file

HIT ICs activate platelets via FcγRIIa. We examined NETs formation by neutrophils in the presence or absence of platelets in the microfluidics system (the purity of isolated neutrophils is shown in Supplementary Fig. 5a–c). In VWF-coated microchannels and in the presence of KKO plus heparin, neutrophils plus platelets resulted in the formation of clots containing platelets, neutrophils, extracellular DNA and CitH3 (Fig. 4a). To explore the events that lead to NETosis in HIT, we next investigated whether platelets activated with HIT IgG ICs induced release of DNA by neutrophils. Washed platelets treated with HIT IgG, purified PF4 and heparin were added to purified autologous neutrophils at a 20:1 platelet to neutrophil ratio (physiological ratio). The production of extracellular DNA, which is indicative of NETosis^34^, was monitored by live cell microscopy. HIT IgG-activated platelets elicited expansive DNA release by neutrophils (Fig. 4b). Induction of NETosis by HIT IgG pre-treated platelets depends on neutrophil–platelet association through P-selectin, since blockage of this interaction with anti-P-selectin (CD62p) or anti-PSGL-1 (CD162) antibodies prevents DNA release (Fig. 4b). Activated platelets have been implicated in NETs formation in other conditions^20,35,36^.

**Fig. 5:**
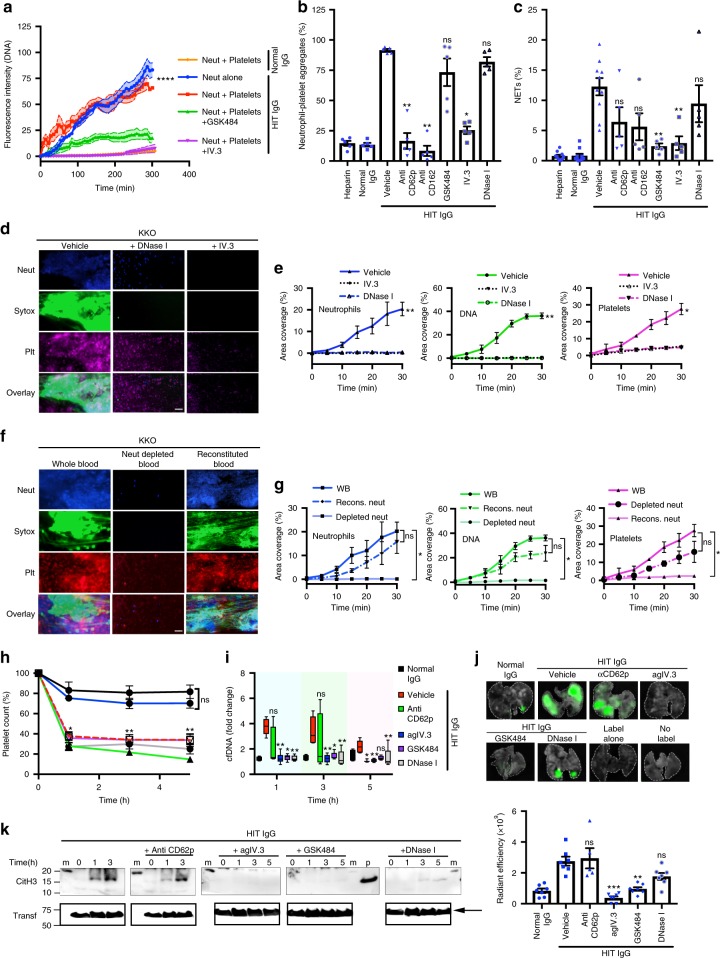
NETosis is required for thrombus formation in HIT. **a** Neutrophils plus platelets were treated with PF4, heparin and normal IgG (*n* = 4, orange), or HIT IgG plus heparin without (*n* = 3, red) or with GSK484 (*n* = 5, green) or IV.3 antibody (*n* = 4, magenta). Treated neutrophils alone are also shown (*n* = 4, blue). The extracellular DNA release determined as in Fig. 4b. Mean ± s.e.m. **b** Quantification of neutrophil–platelet aggregates, mean ± s.e.m. **c** NETs after 5 h treatment with antibodies and inhibitors, mean ± s.e.m. Fluorescent microscopy images of thrombi in microchannels. **d** Blood pre-treated with KKO plus heparin plus vehicle control, DNase I or IV.3. Neutrophils (anti-CD15 AF594, left panel, blue), nucleated cells (Hoechst, middle panel, blue), the extracellular DNA (Sytox green, green) and platelets (anti-CD41 AF647, magenta). Scale bar: 50 μm. **e** Area coverage of neutrophils, DNA and platelets. *n* = 3, mean ± s.d. **f** Whole blood (WB) (left panels), neutrophil (Neut) depleted blood (middle panels) or depleted blood reconstituted (recons) with autologous neutrophils (right panels) incubated with KKO and heparin, treated as described in (**d**). Plt, platelets; Scale bar: 50 μm. **g** Quantification as in (**e**), *n* = 3, mean ± s.d. **h** *FcγRIIa*^*+*^*/hPF4*^*+*^ mice treated with normal (*n* = 5, black) or HIT IgG plus heparin plus vehicle control (*n* = 7, dotted red) or anti-CD62p (*n* = 3, green), agIV.3 antibody (*n* = 8, blue), GSK484 (*n* = 8, magenta) or DNase I (*n* = 8, grey). Mean ± s.e.m. **i** Box-and-whiskers plot of cfDNA in mouse plasma described in (**h**) (*n* = 4 for normal IgG, anti-CD62p; *n* = 5 for DNase I; *n* = 6 for vehicle, IV.3, GSK484). Middle line, bounds of box and whiskers represent the median, 25th to 75th percentiles, and minimum and maximum values, respectively. **j** Mouse lungs imaged as described in (3**b**) and plot of fluorescence intensity. Mean ± s.e.m. **k** Representative western blots of mouse plasma described in (**h**) probed with anti-CitH3 antibody. Arrowhead, CitH3. Arrow, transferrin (transf, loading control). Statistics: (**b**, **c**, **h**, **i**, **j**) Kruskal–Wallis test for comparison of groups with versus without inhibitor. *P*-values adjusted relative to HIT IgG (vehicle). **e**, **g** one-way ANOVA with Tukey’s correction for multiple comparisons. *P < 0.05; **P < 0.01; ***P < 0.001; ****P < 0.0001; ns, not significant. Source data for (**a**, **b**, **c**, **e**, **g**, **h**, **i**, **j**, **k**) are provided as a Source Data file

Unexpectedly, isolated neutrophils resuspended in autologous plasma and incubated with KKO plus heparin formed cell aggregates and released extensive DNA webs and CitH3 when perfused on P-selectin-coated microchannels, demonstrating that under flow conditions neutrophils undergo NETosis upon exposure to ICs formed by KKO (surrogate for HIT IgG), PF4 and heparin in the absence of platelets (Fig. 4c and Supplementary movies 5, 6). Quantitative analysis of percentage area coverage versus time using purified neutrophils and patient’s HIT IgG plus heparin shows deposition of extracellular DNA and neutrophils on P-selectin-coated microchannels (Fig. 4d). This deposition is not observed in the presence of the FcγRIIa-blocking antibody IV.3 or with normal IgG plus heparin (Fig. 4d). These data indicate that HIT ICs can stimulate neutrophils both directly and indirectly via activated platelets, both through FcγRIIa to form extracellular traps. Moreover, fibrin-rich clots can be formed in platelet-depleted *FcγRIIA*^*+*^*/hPF4*^*+*^ mice (Supplementary Fig. 5d–g), suggesting that at least in the animal model of HIT, platelets are not essential for clot formation. As in the microfluidics system experiments shown in Fig. 4c, live cell imaging of neutrophils in the absence of platelets also released extracellular DNA when incubated with HIT IgG, PF4 and heparin in static conditions (Fig. 5a). No significant extracellular DNA release was detected when using normal IgG, PF4 and heparin (Fig. 5a, Supplementary Fig. 6 and Supplementary movies 7, 8), substantiating the previous finding that HIT ICs specifically activate neutrophils and induce NETs formation. The HIT immune complex associates with neutrophils via FcγRIIa since coincubation with the blocking antibody IV.3 prevents KKO– or HIT IgG–neutrophil association (Supplementary Fig. 7a, b). In static conditions, purified neutrophils and platelets released DNA after treatment with HIT IgG, PF4 and heparin, and this was inhibited by IV.3 and by the presence of the NETosis inhibitor (reversible peptidylarginine deiminase 4 (PAD4) inhibitor) GSK484^37^ (Fig. 5a). PF4 alone has been proposed to stimulate NETosis in the presence of platelets^38^; however, we did not observe NETosis after treatment of neutrophils with normal IgG plus PF4 and heparin (Fig. 5a and Supplementary Fig. 6 and Supplementary movies 9, 10).

We then examined the effect of inhibition on NETs formation in whole blood by flow cytometry. Compared with vehicle control, neutrophil–platelet aggregates in whole blood were significantly reduced by antibody blockage of P-selectin (anti-CD62p), PSGL-1 (anti-CD162) or FcγRIIA (IV.3 antibody) (Fig. 5b), while addition of DNase I or GSK484 did not affect cell–cell association (Fig. 5b). Examination of NETosis after 5 h of treatment at 37 °C shows that the PAD4 inhibitor GSK48 and FcγRIIA neutralisation with IV.3 antibody strongly inhibited NETosis (Fig. 5c), while blockage of neutrophil–platelet interaction with anti-CD62p or anti-CD162 had only a modest effect on NETosis. This supports the previous findings (shown in Figs. 4c and 5a) that neutrophils can undergo NETosis following direct stimulation with HIT ICs in the absence of direct neutrophil–platelet interactions.

### NETosis is required for ex vivo thrombus formation in HIT

To dissect the function of NETosis in thrombus formation in HIT, we first examined the role of extracellular DNA on clot formation by pre-digestion of NETs with DNase I. Fresh whole blood was incubated with KKO monoclonal antibody and heparin plus DNase I at 37 °C and perfused into VWF-coated microchannels. The presence of DNase I not only prevented DNA accumulation but also strongly inhibited neutrophil and platelet deposition comparable with the FcγRIIA blocker, IV.3 (Fig. 5d, e). To confirm the role of neutrophils in thrombus formation, we compared the degree of neutrophil, DNA and platelet accumulation in the microfluidics system using whole blood, neutrophil-depleted blood and neutrophil-depleted blood reconstituted with autologous neutrophils. As expected, extensive deposition of neutrophils, extracellular DNA and platelets were observed with whole blood (Fig. 5f). Notably, absence of neutrophils prevented formation of extracellular DNA and markedly inhibited platelet deposition (Fig. 5f). Conversely, addition of autologous purified neutrophils to neutrophil-depleted blood restored neutrophil, DNA and platelet accumulation (Fig. 5f). Accumulation of platelets, neutrophils and DNA in neutrophil-depleted and in reconstituted blood are represented in Fig. 5g. These experiments support the proposal that neutrophils and NETs are required for thrombus formation in HIT, a novel finding that provides important new knowledge of thrombus formation in HIT.

### NETs-dependent thrombosis in HIT

**Fig. 6:**
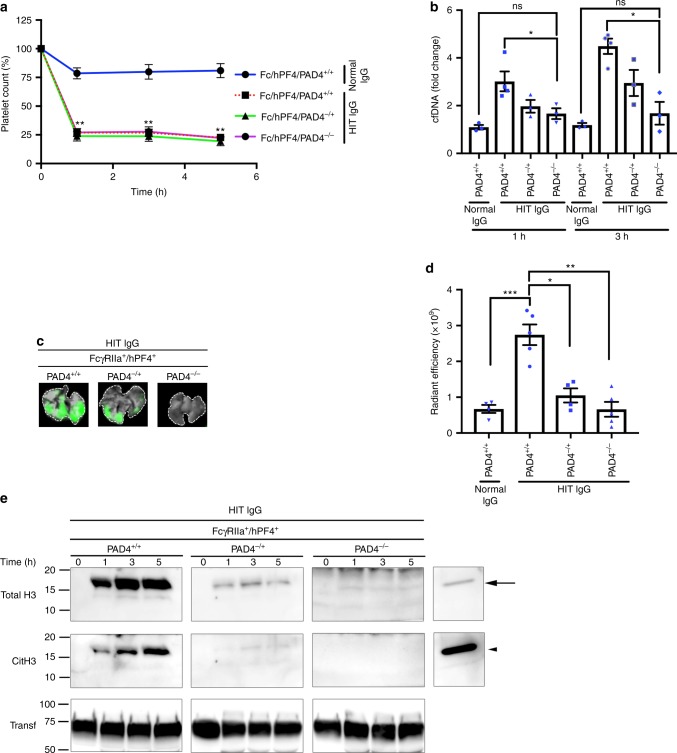
PAD4-deficient mice are protected from HIT IgG-induced thrombosis. **a** *FcγRIIa*^+^/*hPF4*^+^ double transgenic mice wild-type, heterozygous or knockout for *PAD4* (*PAD4*^+/+^, *PAD4*^−/+^ or *PAD4*^−/−^, respectively) were injected intravenously with normal IgG or HIT IgG. Heparin was injected in all cases intraperitoneally at 1 U/g. Platelets were counted at the time points indicated. The percentage of platelets was calculated relative to basal levels. Data are expressed as means ± s.e.m. (*n* = 5). **b** cfDNA in plasma of *FcγRIIa*^*+*^*/hPF4*^*+*^*/PAD4*^*+/+*^ (*n* = 4), *FcγRIIa*^*+*^*/hPF4*^*+*^*/PAD4*^*-/+*^ (*n* = 3) and *FcγRIIa*^*+*^*/hPF4*^*+*^*/PAD4*^−*/−*^ (*n* = 3) mice 1 h and 3 h after normal IgG (*n* = 3) or HIT IgG plus heparin treatment. Mean ± s.e.m. **c** Representative images of mouse lungs for *FcγRIIa*^*+*^*/hPF4*^*+*^*/PAD4*^*+/+*^*, FcγRIIa*^*+*^*/hPF4*^*+*^*/PAD4*^*−/+*^ and *FcγRIIa*^*+*^*/hPF4*^*+*^*/PAD4*^−*/−*^ imaged as described in Fig. 3b. **d** Graphical representation of fold changes in fluorescence intensity (radiant efficiency) in lungs from *FcγRIIa*^*+*^*/hPF4*^*+*^*/PAD4*^*+/+*^ (*n* = 5)*, FcγRIIa*^*+*^*/hPF4*^*+*^*/PAD4*^*−/+*^ (*n* = 4) and *FcγRIIa*^*+*^*/hPF4*^*+*^*/PAD4*^−*/*−^ (*n* = 5) mice described in (**a**). Mean ± s.e.m. **e** Western blots (representative of four independent experiments) of CitH3 in plasma of the transgenic mice described in (**a**) probed with anti-total H3 and anti-CitH3 antibody at the indicated times after treatment with HIT IgG plus heparin. Transferrin was used as a loading control. Arrow denotes total H3. Arrowhead indicates CitH3. Transf, transferrin. Kruskal–Wallis test and *P*-values adjusted by Holm’s Stepdown Bonferroni procedure relative normal IgG were used for statistical analyses. *P < 0.05; **P < 0.01; ***P < 0.001. ns not significant. Source data for (**a**, **b**, **d**, **e**) are provided as a Source Data file

To determine the role of NETosis in thrombus formation in vivo, we induced the HIT condition in *FcγRIIA*^*+*^*/hPF4*^*+*^ mice in the presence of DNase I, anti-P-selectin antibody (anti-CD62p) and the NETs inhibitor GSK484, which is known to inhibit NETosis in mouse neutrophils^37,39^. Effector deficient IV.3 (aglycosylated IV.3, agIV.3) was also used as a HIT-blocking control (unmodified IV.3 cannot be used in mice expressing FcγRIIA because it leads to thrombocytopenia and anaphylactic reactions^40^). Thrombocytopenia (Fig. 5h) and decrease in body temperature (Supplementary Fig. 8a) were not specifically affected by the presence of GSK484, DNase I or anti-CD62p antibody. The extent of cfDNA in circulation was reduced by all inhibitors, except anti-CD62p, 1 h and 3 h after treatment (Fig. 5i). Unlike the effect on platelet count, inhibition of NETs formation by GSK484 caused a dramatic reduction in thrombus deposition in mouse lungs (Fig. 5j). Unlike GKS484, and in agreement with the hypothesis that neutrophil–platelet interactions are dispensable for HIT–IgG-induced NETosis, mice treated with anti-CD62p antibody did not show inhibition of thrombus formation (Fig. 5j). As expected, neutralization of FcγRIIA by agIV.3 completely abolished thrombus deposition in the lungs. Inhibition by DNase I was less effective, perhaps due to protection of the NETs by HIT ICs^27^. Moreover, CitH3 was absent in the plasma of GSK484- or agIV.3-treated mice (Fig. 5k). The detected levels of CitH3 were not affected by the presence of anti-CD62p antibody (Fig. 5k), while DNase I treatment reduced the levels of CitH3 in plasma (Fig. 5k). Collectively, these results indicate that inhibition of platelet and neutrophil activation by blockade of FcγRIIA or inhibition of NETs formation with GSK484 is sufficient to prevent thrombus formation in vivo. Nuclear decondensation is driven by PAD4, therefore PAD4-deficient mice represent a robust tool to study NETosis in vivo. To ascertain the role NETosis in HIT, we used PAD4-deficient *FcγRIIA*^*+*^*/hPF4*^*+*^ mice (*FcγRIIA*^*+*^*/hPF4*^*+*^*/PAD4*^*−/−*^). Absence of PAD4 in these mice was determined by western blot (Supplementary Fig. 8b). In addition, the number of neutrophils and platelets in *FcγRIIA*^*+*^*/hPF4*^*+*^*/PAD4*^*−/+*^ and *FcγRIIA*^*+*^*/hPF4*^*+*^*/PAD4*^*−/−*^ mice were comparable with *FcγRIIA*^*+*^*/hPF4*^*+*^*/PAD4*^*+/+*^ animals (Supplementary Fig. 8c). PAD4 deficiency did not protect mice from HIT IgG-induced thrombocytopenia (Fig. 6a), which is consistent with findings using GSK484. The presence of cfDNA, however, was reduced in *FcγRIIA*^*+*^*/hPF4*^*+*^*/PAD4*^*−/−*^ mice (Fig. 6b) and the extensive thrombus deposition observed in lungs from WT mice (*FcγRIIA*^*+*^*/hPF4*^*+*^*/PAD4*^*+/+*^*)* was absent in PAD4 deficient mice (Fig. 6c, d). In contrast to the strong circulating levels of total H3 and CitH3 in WT mice, markedly low levels of total H3 and no CitH3 were detected in *FcγRIIA*^*+*^*/hPF4*^*+*^*/PAD4*^*−/−*^ animals (Fig. 6e). Absence of PAD4 considerably protected treated mice from the drastic decrease in body temperature and other systemic reactions observed in WT mice (Supplementary Fig. 8d). Of note, isolation of mouse peripheral blood mononuclear cells by gradient centrifugation demonstrated the presence of LDGs only in *FcγRIIA*^*+*^*/hPF4*^*+*^*/PAD4*^*+/+*^ mice (Supplementary Fig. 8e). These cells were virtually absent in *FcγRIIA*^*+*^*/hPF4*^*+*^*/PAD4*^*−/+*^ and *FcγRIIA*^*+*^*/hPF4*^*+*^*/PAD4*^*−/−*^ mice (Supplementary Fig. 8e, middle and lower panels). Thus, our data demonstrate that HIT IgG activates neutrophils to induce NETosis and that NETs are essential for thrombus formation in HIT.

## Discussion

Thrombosis is the most critical aspect of HIT that contributes to morbidity and mortality^41,42^. Catastrophic thrombotic complications such as limb gangrene and mortality rate have not decreased significantly despite treatment with non-heparin anticoagulants, such as argatroban and lepirudin^7,8^. There is an unmet clinical need to explore new mechanisms involved in the development of thrombosis in HIT.

In this study, we have shown the occurrence of NETosis in HIT by demonstrating markers of neutrophil activation and NETosis (increased MPO and elastase, extracellular DNA and CitH3) in HIT patients’ plasma, and activated and NETting neutrophils and increased neutrophil–platelet aggregates in blood of patients with HIT. These NETosis markers were also present in the plasma of *FcγRIIa*^*+*^*/hPF4*^*+*^ mice when the HIT condition was recreated by administration of HIT IgG antibodies and heparin. In addition, neutrophils with nuclear decondensation (characteristic of NETosis) together with CitH3 and extracellular DNA were observed in platelet-rich thrombi in the lungs of these mice with HIT. These markers of NETosis were also detected in thrombi generated ex vivo under flow conditions (microfluidics) as well as static conditions (live cell microscopy and flow cytometry).

Importantly, we found that HIT ICs can directly activate neutrophils by two pathways: (1) direct activation of neutrophils via FcγRIIa, and (2) by activation of platelets via FcγRIIa, followed by the interaction and activation of neutrophils via P-selectin and PSGL-1. The second pathway is not essential for HIT ICs to generate NETs, as anti-CD62p and anti-CD162 antibodies did not block NETosis induced by HIT ICs. The P-selectin/PSGL-1 interaction is not the only molecular mediator of neutrophil/platelet association^43^. However, in our experiments, blockage of CD62p or CD162 was sufficient to markedly inhibit neutrophil/platelet interaction induced by HIT antibodies and to prevent DNA release by neutrophils in the presence of HIT ICs-activated platelets. This suggests that in HIT this is likely to be the main form of interaction, but in other situations other pathways may be involved.

The results presented here also show that the activation of neutrophils and the formation of NETs are critical for thrombus generation in HIT. In the HIT murine model using *FcγRIIa*^*+*^*/hPF4*^*+*^ mice, treatment with DNase I decreased thrombus formation while administration of PAD4 inhibitor GSK484 blocked NETosis and also markedly reduced thrombus formation. PAD4-deficient mice failed to develop thrombi when they were injected with HIT antibodies and heparin. Furthermore, digestion of DNA prior to perfusion of whole blood into microchannels or neutrophil depletion abrogates platelet and neutrophil accumulation. These findings are consistent with reports that NETs fuel thrombus formation, trap platelets and stimulate fibrin deposition^22^. In HIT, thrombocytopenia can occur alone or with thrombosis^44^. Our results indicate that thrombocytopenia and thrombosis are two separable processes, but both are consequences of HIT immune complex activity. Binding of the complexes to platelets via FcγRIIa leads to thrombocytopenia which occurs even in the absence of NETosis (Fig. 5h and Fig. 6a). The thrombocytopenia in HIT^42^ is likely to be due to removal of HIT immune complex-coated platelets by macrophage phagocytosis^45^. In contrast, thrombosis in HIT requires neutrophil activation and, more specifically, the formation of NETs. These observations are contrary to the current prevailing concept that thrombosis is mainly driven by platelet activation. In fact, fibrin-containing clots were found in lungs of platelet-depleted mice treated with HIT IgG and heparin. These findings have clinical significance since effective treatment of thrombosis in HIT is still an unresolved challenge^8,46^. This demonstration that thrombosis in HIT is mediated by NETosis may, in part, explain why anticoagulation alone is not sufficient for treatment of thrombosis. Consequently, inhibition of NETosis represents a potential intervention for the prevention and reduction of thrombosis in HIT.

Recently, Gollomp et al.^27^ also studied NETosis in HIT and concluded that neutrophils and NETosis may be implicated in the pathology of HIT. Our findings further those observations by providing strong evidence that HIT IC-induced NETosis is essential for thrombus development in HIT. These investigators showed in a laser injury model using *FcγRIIa*^*+*^*/hPF4*^*+*^ mice that neutrophils are present in venular thrombi in the presence of KKO^27^. The size of the thrombi appears to depend on NETosis, since clots were smaller in *PAD4*^−*/*−^ mice or in animals pre-treated with DNase I^27^. Our results indicate not only the presence of neutrophils in HIT IC-induced thrombi, but also abundant NETs, CitH3 and MPO. In addition, we found that thrombus formation induced by HIT or KKO ICs was substantially abolished by inhibition of NETosis with GSK484.

In conclusion, our data show that in HIT, thrombocytopenia and thrombosis are separable events (see diagram in Supplementary Fig. 9) and that HIT ICs induce the formation of neutrophil–platelet aggregates, indirectly activate neutrophils and promote NETosis which is an essential step in the thrombotic process. Importantly, NETosis is also induced by HIT ICs by direct activation of neutrophils through FcγRIIa without the need for platelet participation. Formation of thrombi in HIT mice is inhibited by blockage of NETosis. Consistent with this, we found that PAD4-deficient *FcγRIIa*^*+*^*/hPF4*^*+*^ mice are protected from HIT antibody-induced thrombosis. We conclude that neutrophil activation and the resulting NETosis are major drivers of thrombosis in HIT, and the inhibition of NETs could be exploited therapeutically. Thus, anti-NETs-targeted therapy alone or preferably in conjunction with non-heparin anticoagulants may be more efficacious and would prevent disastrous thrombotic consequences, such as limb amputation and death, and significantly improve patient outcomes.

## Methods

### Human samples

Plasma was collected from patients with a clinical diagnosis of HIT, and positive for laboratory tests (HIT ELISA and serotonin release assay). The diagnosis of HIT was made according to the criteria outlined previously^1^. Eighteen of the 21 patients with HIT had arterial/venous thrombosis; three had only thrombocytopenia without thrombosis (isolated HIT). The study was approved by the University of New South Wales’ Human Research Ethics Committee (HC12093) and the South Eastern Sydney Local Health District Human Research Ethics Committee (17/349 LNR/18/POWH/45). Blood and plasma from all donors were obtained with informed consent. Plasma samples were stored at −80 °C until use.

### Antibodies

Total HIT IgG was purified from patients’ plasma with Protein G Agarose (Roche Mannheim, Germany). Resin (2 ml for 10 ml of plasma) was packed into a chromatography column (1.5 cm × 10 cm; Bio-Rad, Hercules, CA, USA), and the purification procedure was conducted with AKTA purifier chromatography system (GE Healthcare). The eluted peak fractions were pooled and concentrated using ultracentrifugal unit (10 kDa) (Millipore, Billerica, MA, USA). Activity of purified HIT IgG was determined by platelet aggregation and serotonin release assays.

### Cell culture and antibody purification

Monoclonal antibody-producing hybridoma cells for IV.3 were obtained from ATCC^®^ (clone HB-217^TM^) and for KKO were kindly provided by Prof. Gowthami Arepally (Duke University). Cells were cultured in DMEM medium containing 10% FBS. The culture was maintained in a humidified incubator at an aeration of 5% CO_2_ at 37 °C. Cells were cultured for 24 h in serum-free DMEM before collection of antibody-containing supernatant. Antibodies were purified by protein G sepharose affinity chromatography.

### Reagents

Reagents used for NETosis experiments were purchased with low endotoxin levels (i.e. < 0.01 EU/µg of protein) e.g. Ultra-LEAF purified CD62p (148308, BioLegend). Endotoxin levels were also confirmed with Pierce LAL Chromogenic Endotoxin Quantitation Kit (88282, Thermo Scientific).

### HIT mouse model

Double transgenic *FcγRIIA*^*+*^*/hPF4*^+^ mice were generated by crossing transgenic mice expressing the R^131^ isoform of human FcγRIIA with mice transgenic for human PF4^29^. These mice were generously provided by Dr Steven McKenzie (Thomas Jefferson University).

*PAD4*^−*/*−^ mice were produced by the Mouse Engineering Garvan/ABR (MEGA) Facility (Moss Vale and Sydney, NSW, Australia) by CRISPR/Cas9 gene targeting in C57BL/6J mouse embryos following established molecular and animal husbandry techniques^47^. A single-guide RNA (sgRNA) was designed to target within exon 2 of mouse *Padi4* gene (target with protospacer-associated motif underlined: CCGAAGGTTGTGTAGCCCTTAGG) and coinjected with polyadenylated Cas9 mRNA into C57BL/6J zygotes. Microinjected embryos were cultured overnight and introduced into pseudo-pregnant foster mothers. Pups were screened by PCR and Sanger sequencing of ear-punch DNA, and a founder mouse identified that carried a 10 bp frame-shift deletion in exon 2. The targeted allele was maintained by breeding on a C57BL/6J background. *PAD4*^*−/−*^ mice were crossed with *FcγRIIA*^*+*^*/hPF4*^*+*^ mice to generate *FcγRIIA*^*+*^*/hPF4*^*+*^*/PAD4*^−*/−*^ animals. Multiple mice between 8 and 12 weeks of age were randomly selected and analysed as biological replicates. All analyses were performed unblinded except for platelet counts and histology analyses. No power calculations were done for animal experiments. All animal experiments were approved by the Animal Care and Ethics Committee of the University of New South Wales.

### Quantification of NETosis markers

Plasma levels of cfDNA were measured using a fluorometric assay for double-stranded DNA Quant-iT PicoGreen dsDNA Assay kit (Invitrogen) following the manufacturer’s instructions. Plasma levels of citrullinated histone H3, human myeloperoxidase, neutrophil elastase and VWF were determined by ELISA with Citrullinated Histone H3 (Clone 11D3) ELISA Kit (Cayman Chemicals), Human Myeloperoxidase Quantikine ELISA Kit (R&D Systems), Human PMN Elastase Platinum ELISA (eBioscience, San Diego, CA) or by in-house ELISA using sheep anti-human VWF (Abcam, ab11713) for coating followed by detection with polyclonal rabbit anti-human VWF-HRP (Dako, P0226), respectively.

### Cell isolation and in vitro NETosis

EDTA-anticoagulated blood was used to isolate neutrophils using EasySep Direct Human Neutrophil Isolation Kit (19666; StemCell Technologies) following the manufacturer’s instructions. Neutrophils were depleted from whole human blood with CD15 microbeads from Miltenyi Biotech following the manufacturer’s instructions. Purity was assessed by flow cytometry. Washed platelets were prepared from citrate-anticoagulated blood^48^. For live cell imaging, cells were treated with purified HIT IgG or control IgG, purified PF4 and heparin. HIT IgG-stimulated cells were labelled with CellTracker™ Deep Red dye or CellTracker™ Orange CMTMR Dye (Invitrogen). Purified autologous neutrophils were reacted with platelets at a 1:20 ratio. The total DNA and extracellular DNA were stained with Hoechst 33342 (145333, Sigma) and Sytox^®^ Green nucleic acid stain (Invitrogen), respectively. Inhibitors included: IV.3, GSK484 (Cayman chemicals), anti-CD62p (304902, BioLegend), anti-CD162 (328802, BioLegend) and DNase I (18047019, Invitrogen).

### Ex vivo microfluidics reconstitution of HIT

Vena8 Fluoro + ™ biochip microchannels were coated with VWF (Haematologic Technologies United BioResearch Products Pty Ltd) or P-selectin (R&D systems) at a concentration of 200 μg/ml at 4 °C overnight. Microchannels were washed with PBS then blocked with PBS/1% BSA for 30 min. Citrated whole blood was diluted 1:2 with PBS, supplemented with purified IgG (HIT IgG 3 mg/ml, normal IgG 3 mg/ml, KKO 1 mg/ml) and heparin (0.5 U/ml) and incubated for 1.5 h at 37 °C. Before perfusion, the blood was stained with combinations of the following reagents: Hoechst 33342 (3 μg/ml), Sytox green (0.3 μM), anti-CD41 Alexa 647 (15 μg/ml), anti-CD15 Alexa 647 (15 μg/ml), anti-fibrin Alexa 594 (30 μg/ml), anti-CitH3 Alexa 594 (30 μg/ml). The assay was performed at 37 °C in a Venaflux microfluidics device (Cellix Ltd. Dublin, Ireland) in the absence and presence of inhibitors (DNase I (160 U/ml), IV.3 (20 μg/ml) at a fluid shear stress of 67 dyne/cm^2^ (shear rate 1500 s^−1^) for up to 30 min. Flow chambers were mounted on a fluorescent microscope (Zeiss Axio Observer.A1) and fluorescence images from different microscopic fields were captured in real time with a Q-Imaging EXi Blue™ camera (Q-Imaging, Surry, BC, Canada) driven by Venaflux software (Cellix Ltd. Dublin, Ireland). Accumulation of DNA, platelets and neutrophils was measured by calculating the surface area coverage with Image-Pro Premier 9.1 software (Media Cybernetics, Inc, Rockville, MD, USA). Selected channels were perfused with 2% paraformaldehyde to fix the samples for subsequent imaging by confocal microscopy.

### IVIS spectrum CT imaging

Following euthanasia, the thoracic cavity of the mouse was exposed through a midline sternotomy. Lung perfusion was conducted using a 25-gauge needle with 1.5 ml PBS followed by 0.5 ml 10% formalin (HT501128; Sigma). The lungs were gently teased from their pleural adhesions and placed into 10% formalin. Fluorescence in the lungs was imaged within 2 h of harvesting using the IVIS Spectrum (Perkin Elmer) with 570/680 nm (background) and 640/680 filter sets. Image Math in Living Image 4.5.5 software (Perkin Elmer) was used to calculate fluorescence in radiant efficiency.

### Histology

Fixed lungs were paraffin embedded, sectioned at 4 microns and mounted onto slides. Slides were subjected to H&E or Carstairs staining. For immunohistochemistry, slides were deparaffinized and rehydrated, followed by 20-min heat-induced antigen retrieval with Tris/EDTA pH 9. The slides were washed with TBS/0.025% Triton X-100 then blocked with 10% donkey or goat serum with 1% BSA in TBS. Slides were probed with the following primary antibodies: anti-CitH3 (ab5103; Abcam), anti-Ly6G (127602; Biolegend) and anti-MPO (ab9535; Abcam). The secondary antibodies used were donkey anti-rabbit IgG, Alexa 647 (ab150075, Abcam), AffiniPure donkey anti-rabbit IgG, Alexa 488 (711-545-152; Jackson ImmunoResearch Laboratories) or goat anti-rat IgG, Alexa 594 (ab150160; Abcam). Glass coverslips were mounted onto the slides using Vectashield antifade mounting medium with DAPI (H-1200; Vector Laboratories) and imaged with fluorescence or confocal microscopy.

### Confocal and fluorescence microscopy

Live or fixed cells (isolated neutrophils with or without platelets, and thrombi) in biochip microchannels or eight-well Nunc Lab-Tek II chambers and lung sections were stained and imaged with the objectives described in the corresponding figure legends with a confocal laser-scanning microscope (Leica TCS SP8) running Leica’s LAS X software. Fluorescent images were taken with the objective indicated in the corresponding figure legend with a Zeiss Axio Vert.A1 microscope and Zen software version 1.1.1.0. Transmission images were taken with a Zeiss Axioskop microscope running Zen software version 2.3.64.0.

### Flow cytometry

Citrated blood freshly collected from HIT patients and healthy donors was diluted in PBS and stained with anti-CD15 (Alexa 647, BD 562369), anti-CD16 (APC-Cy7, BD 557758), anti-CD41a (V450, BD 561425), anti-citrullinated histone H3 (ab5103), anti-MPO (PE, BD 341642) and Affinipure donkey anti-rabbit (Alexa 488, Jackson ImmunoResearch Laboratories 711545152) antibodies. Samples were fixed with paraformaldehyde and analysed by flow cytometry (CantoII, BD Bioscience). Citrated blood from healthy donors was also treated in vitro with HIT or control antibodies, labelled and analysed as described above. Fluorescent counting beads (Invitrogen C36950) were used to derive absolute cell numbers by flow cytometry following the manufacturer’s instructions. Platelet counts from animal experiments were determined by the number of events acquired in 60 s. LDG and monocytes from human and mouse experiments isolated using Histopaque separation media (Sigma)^49^ were identified using anti-CD15 (Alexa 647, BD 562369), anti-CD16 (APC-Cy7, BD 557758, anti-CD14 (V500, BD 561391), anti-Ly6G (V450, BD560603) and anti-CD11b (APC, BioLegend 101212).

### Western blotting

Albumin was depleted from plasma samples as described in the Pierce™ Albumin Depletion Kit protocol (85160, Thermo Scientific). Proteins were separated by SDS-PAGE on 4–20% polyacrylamide gels (Bio-Rad) and stained with Coomassie Blue or Ponceau S. Western blot analyses^50^ were carried out as follows: membranes were probed with the primary antibodies: anti-CitH3 (dilution 1:1000; ab5103; Abcam), anti-transferrin (dilution 1:2000; ab82411; Abcam), anti-Histone H3 antibody (1:2000; total H3; ab1791; Abcam), anti-PAD4 (1:1000; ab214810; Abcam) and anti-β-actin (1:2000; AC-15; Sigma). The secondary antibodies used were anti-rabbit HRP (1:2000; P0260; DAKO, Denmark) or anti-mouse HRP (1:1000; P0447; DAKO, Denmark). Signals were detected using SuperSignal West Dura Extended Duration Substrate (ThermoFisher Scientific) and an ImageQuant LAS 4000 imager (GE Healthcare). Uncropped scans of western blots are provided in the Source Data file.

### Statistics

For animal experiments, mice between 8 and 12 weeks of age were randomly selected and analysed as biological replicates. For in vitro and ex vivo experiments, blood samples from different healthy donors were used as biological replicates. All analyses were performed unblinded, except for platelet counts and histology. Normality of the data was tested using the Shapiro–Wilk normality test. Nonparametric data with multiple comparisons were analysed by Kruskal–Wallis one-way analysis of variance followed by Holm’s Stepdown Bonferroni procedure for adjusted *p*-values. The Mann–Whitney *t* test was used for comparison between two groups. Data with normal distribution were analysed by one-way ANOVA with Dunnett’s post-test or Tukey’s correction for multiple comparisons as described in the figure legends. Statistical significance was set at *p* < 0.05. Data were analysed using Prism 7.0 software (GraphPad, La Jolla, CA).

## Supplementary information

Supplementary information

Supplementary Movie 1

Supplementary Movie 2

Supplementary Movie 3

Supplementary Movie 4

Supplementary Movie 5

Supplementary Movie 6

Supplementary Movie 7

Supplementary Movie 8

Supplementary Movie 9

Supplementary Movie 10

dataset

## Data Availability

The data that support the findings of this study are available from the corresponding author upon reasonable request. All data generated or analysed during this study are included in this published article (and its Supplementary information files). The source data underlying all relevant figures are provided as a Source Data file. A reporting summary for this article is available as a supplementary information file.
