# Supplementary material for: Neutrophil activation and NETosis are the major drivers of thrombosis in heparin-induced thrombocytopenia
Source: Nat Commun. 2019 Mar 21;10:1322. doi: 10.1038/s41467-019-09160-7 (PMC6428879; doi:10.1038/s41467-019-09160-7)
Supplement: Supplementary file 1 — Supplementary information [file 41467_2019_9160_MOESM1_ESM.pdf]

Neutrophil activation and NETosis are the major drivers of thrombosis in heparin-induced thrombocytopenia

Perdomo et al.

Supplementary Information

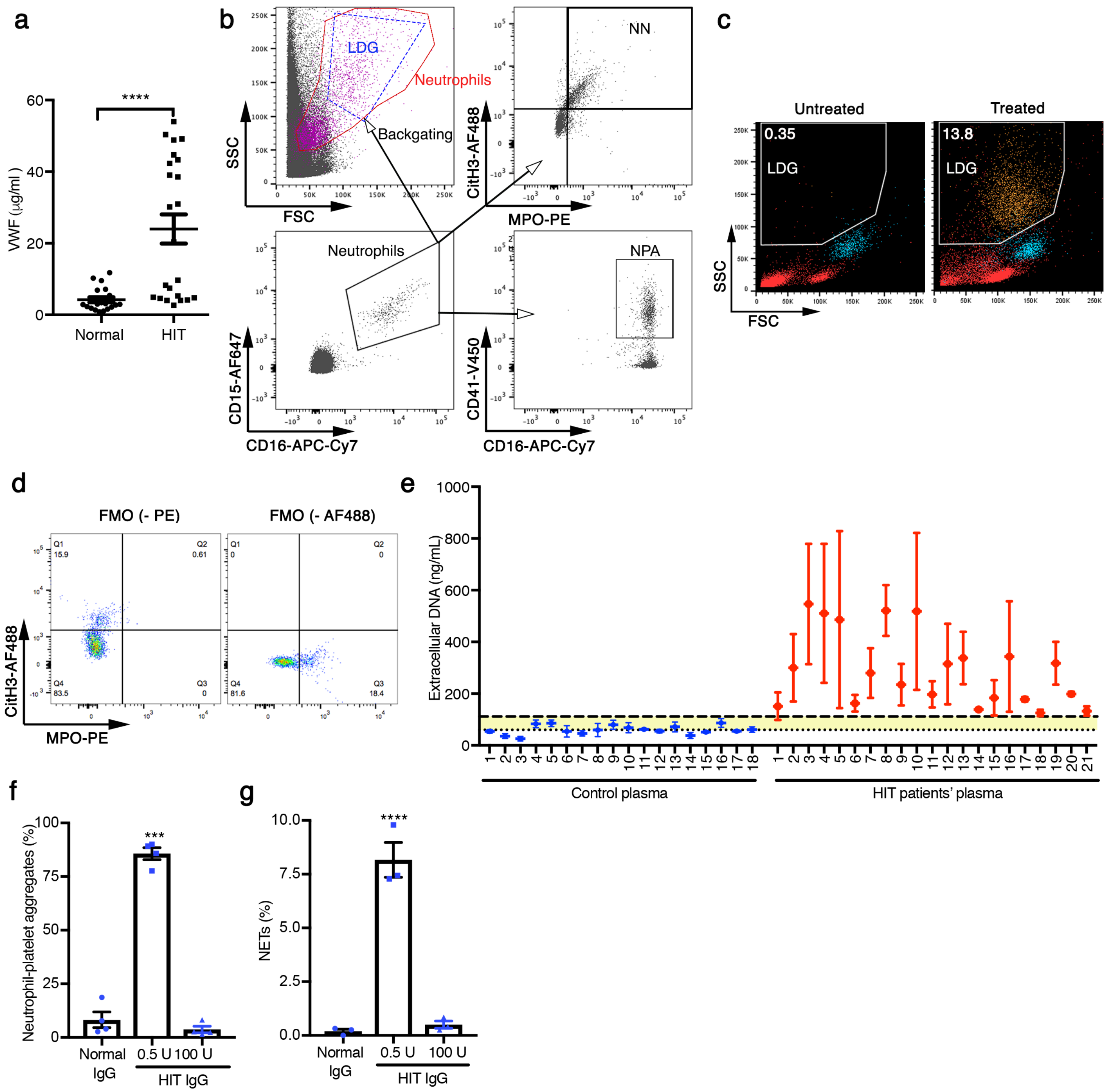

Supplementary Figure 1. (a) VWF concentration in HIT patients' plasma (n=23) relative to normal controls (n=20) (mean ± s.e.m) was determined by ELISA. **(b)** Gating strategy. Neutrophils were identified in whole blood as $CD15^+/CD16^+$ events Backgating onto the whole blood scatter plot shows neutrophils (red gate) and LDGs (blue gate). Within $CD15^+/CD16^+$ population, NETting neutrophils and neutrophil/platelet aggregates were identified as $CitH3^+/MPO^+$ or $CD41^+/CD16^+$ events, respectively. This strategy was used for data shown in Fig. 1f, Fig. 2a, Fig. 5b,c and Supplementary Fig. 1e,f. LDGs, low density granulocytes; NN, NETting neutrophils; NPA, neutrophil-platelet aggregates. **(c)** Untreated (left panel) and HIT IgG-treated (right panel) peripheral blood mononuclear cells. Low density granulocytes (LDGs) are absent (< 1%) in untreated blood but they are enriched in HIT IgG-treated samples. Monocytes ($CD14^+$, blue) and neutrophils ($CD14^-CD15^+CD16^+$, orange) were backgated and are shown within the total PBMC population (red). **(d)**, Fluorescence minus one (FMO) controls for AF488 and PE for fresh blood samples treated with HIT IgG plus heparin. **(e)** DNA release by neutrophils treated with patients' or normal plasma plus heparin (normal plasma controls, n=18, blue dots; HIT patients' plasma, n=21, red dots). Mean ± s.d of three independent experiments. Yellow shaded area indicates 3 s.d above the control samples mean. **(f, g)** Supra therapeutic heparin concentration (100 U/ml) inhibits **(f),** neutrophil-platelet aggregation and **(g)**, NETs formation. Mean ± s.e.m. ***P < 0.001****P < 0.0001. Statistics, for (a) Mann-Whitney test; for (f.g) one-way ANOVA with Tukey's correction for multiple comparisons. Source data for (a, e, f, g) are provided as a Source Data file.

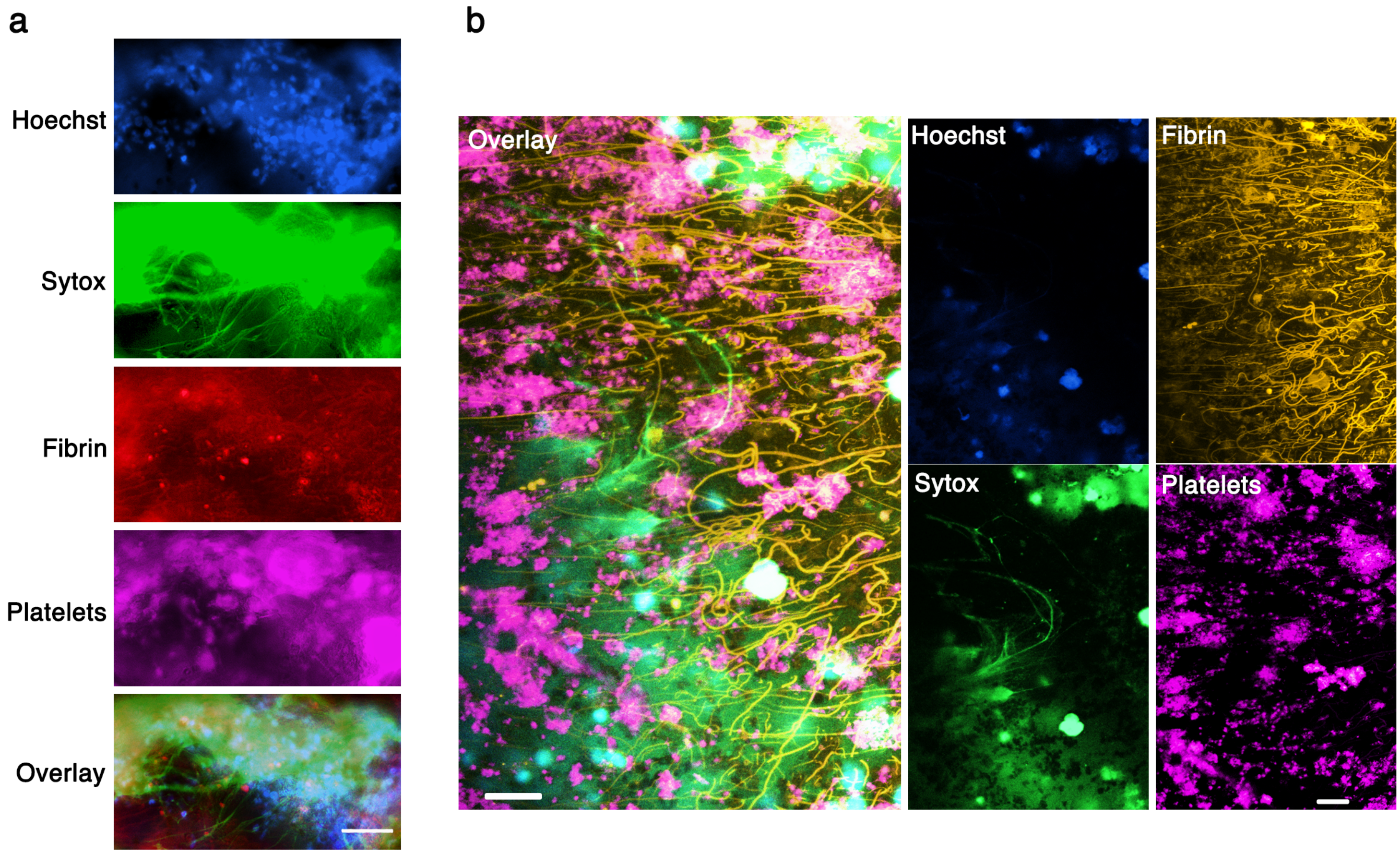

Supplementary Figure 2. (**a**) Whole blood was incubated with HIT IgG and heparin at 37°C for 1.5h and perfused into VWF-coated microchannels at a shear stress of 67 dynes/cm^2 at 37°C for up to 30 min. Cells were stained with Hoechst (blue), extracellular DNA with cell impermeable Sytox green (green), fibrin with anti-fibrin Alexa 594 (red) and platelets with anti-CD41 Alexa 647 (magenta). Experiments were conducted at 37°C in VWF-coated microchannels at arteriole shear stress.Scale bar 50 μm.
(**b**) NETs are present in thrombi formed ex vivo in a microfluidics system. Representative images taken by confocal microscopy of thrombi formed after perfusion of citrated human blood pre-treated with HIT IgG and heparin. Scale bar 20 μm.

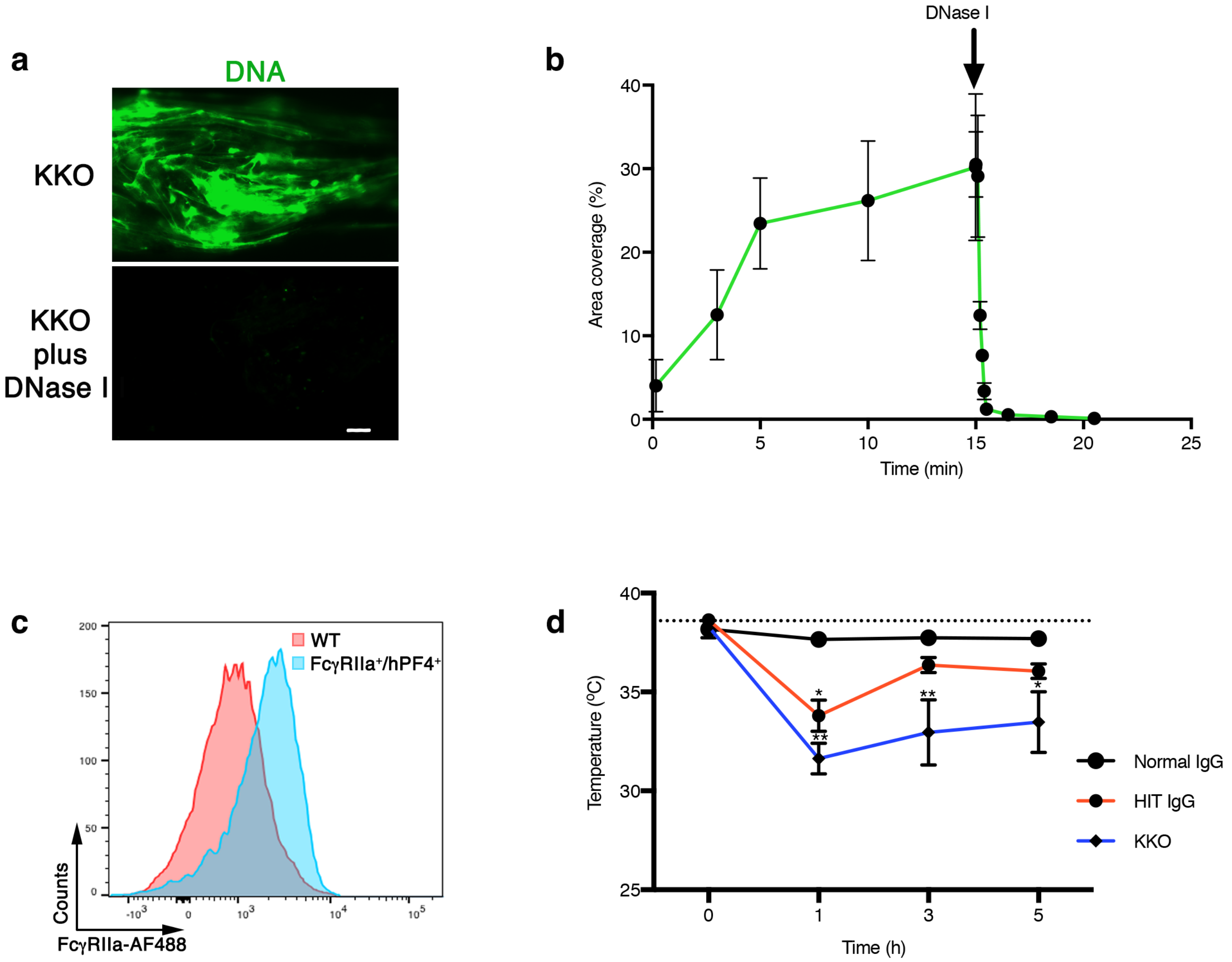

Supplementary Figure 3. (a) Whole blood was incubated with KKO and heparin at 37ºC for 1.5h and perfused into VWF-coated microchannels at a shear stress of 67 dynes/cm^2 at 37ºC for up to 15 min (top panel). DNase I was added to the microchannel reservoir and the perfusion was continued for 5 min (bottom panel). Scale bar, 50µm. **(b)** Percentage area coverage of VWF-coated surfaces versus time for extracellular DNA in DNase I-treated samples. DNase I was added after 15 min of perfusion as indicated by the arrow. Percentage coverage area determinations were calculated for the times indicated by the markers. Mean ± s.d are shown (n=3). **(c)** Expression of FcγRIIa on neutrophils of *FcγRIIA$^+$/hPF4$^+$* mice. Neutrophils were enriched from bone marrow of WT or *FcγRIIA$^+$/hPF4$^+$* mice using EasySep™ Mouse Neutrophil Enrichment Kit (Stemcell Technologies™). Neutrophils (Ly6G$^+$ cells) were stained with IV.3 antibody and anti-mouse IgG F(ab')$_2$-AF488. Red histogram, WT neutrophils; blue histogram *FcγRIIA$^+$/hPF4$^+$* neutrophils. **(d)** Rectal temperature after treatment of *FcγRIIa$^+$/hPF4$^+$* mice with HIT IgG, KKO or normal IgG (n=5 for normal IgG and KKO; n=4 for HIT IgG). Dotted line represents the mean body temperature of mice before treatment (38.6ºC, n=60). Mean ± s.e.m. Kruskal-Wallis test adjusted for multiple comparisons by Holm's stepdown Bonferroni procedure. *P <0.05; **P < 0.01. Source data for (b, d) are provided as a Source Data file.

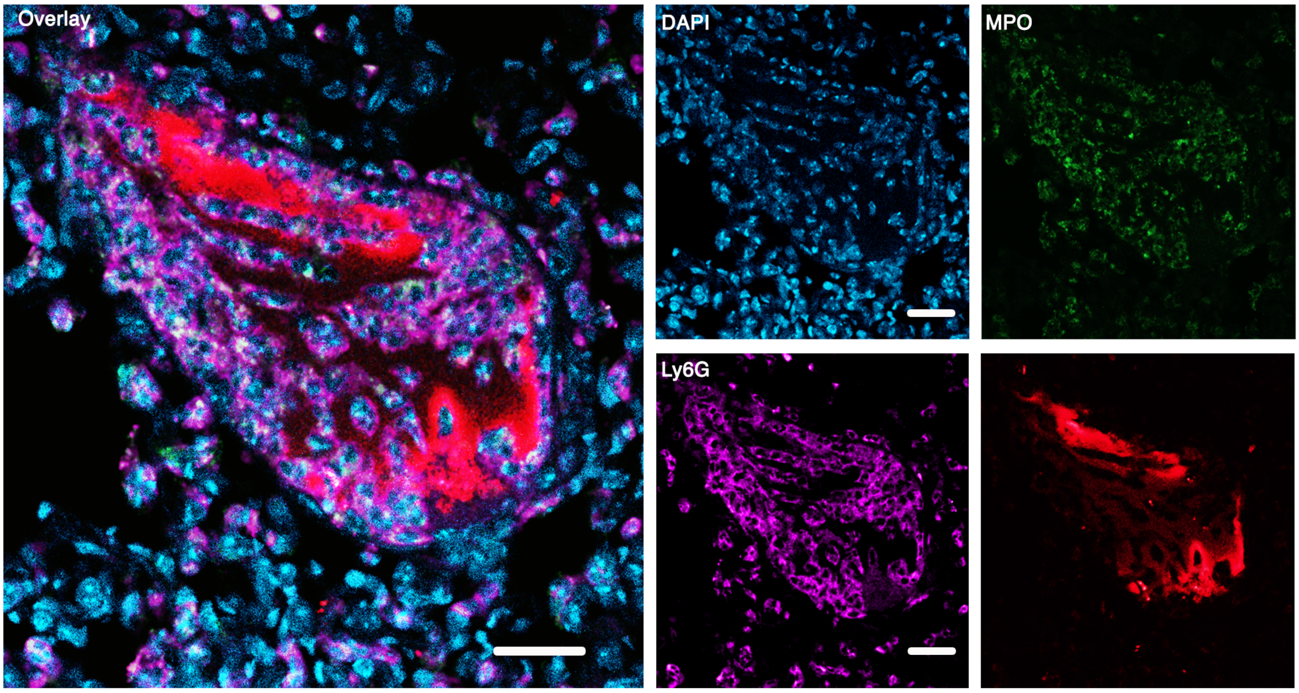

Supplementary Figure 4. Fibrinogen labelled with Hylite-647 was injected into mice to allow deposition of Hylite-647 labelled fibrin. Animals were treated with HIT IgG plus heparin and lung sections from these mice were imaged by confocal microscopy with a 40X water objective. The clots show abundant fibrin content (red), MPO (green) and neutrophils (Ly6G^{+} cells, magenta). Cell nuclei were stained with DAPI (blue). Scale bar 20μm.

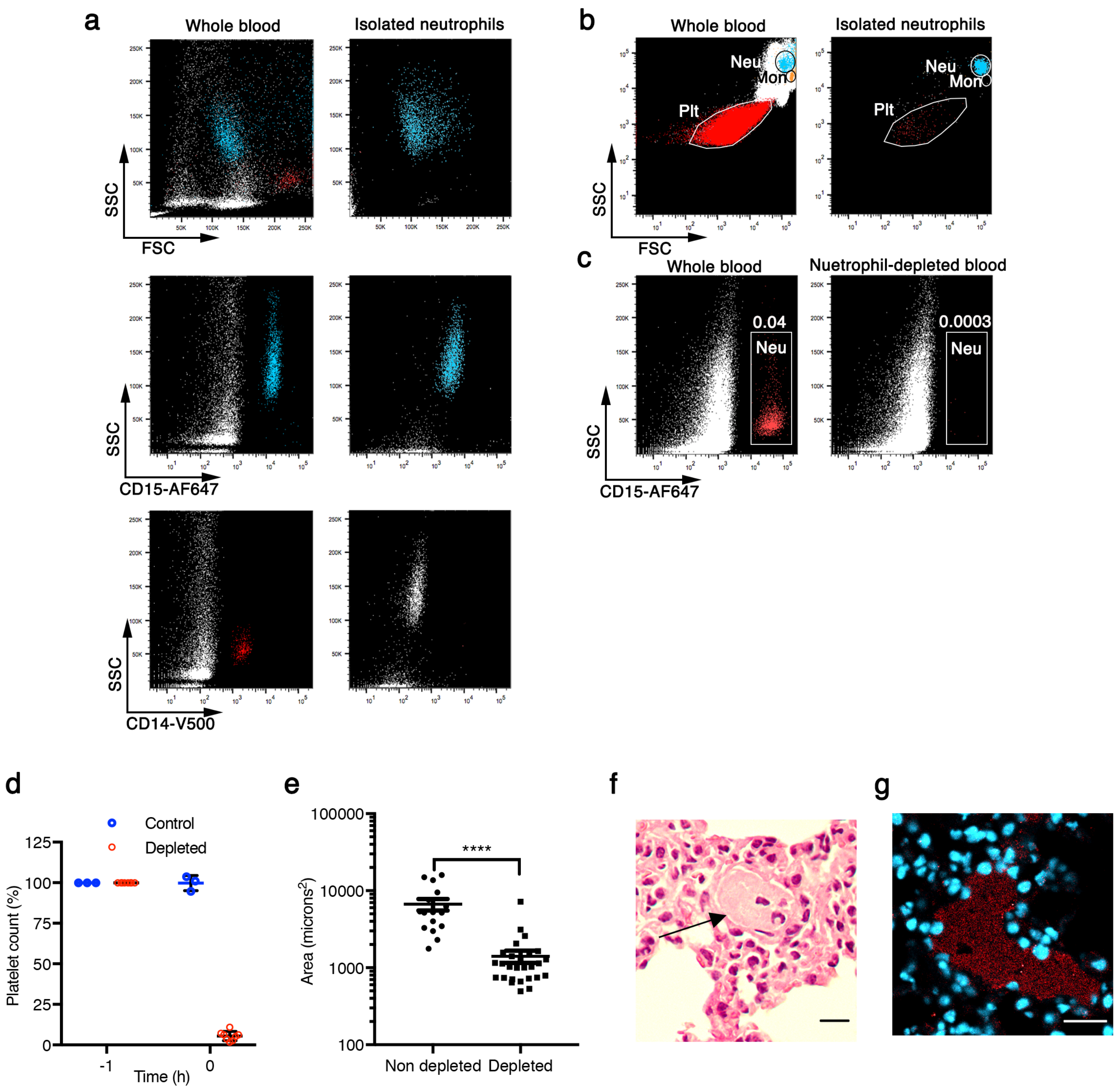

Supplementary Figure 5. (a) Representative flow cytometry plots of neutrophils (CD15$^+$ cells, blue) and monocytes (CD14$^+$ cells, red). Top panels show side scatter versus forward scatter of whole blood (left panel) or isolated neutrophils (right panel) backgated for monocytes and neutrophils. Middle panels show neutrophils and lower panels show monocytes. No monocytes are present in the purified neutrophil fraction. **(b)**, Representative flow cytometry plots of neutrophils (CD15$^+$ cells, blue), monocytes (CD14$^+$ cells, orange) and platelets (CD41$^+$ cells, red). Dotplots show side scatter versus forward scatter of whole blood where neutrophils, monocytes and platelets are indicated. Residual platelets in the isolated neutrophil population represents 0.004% of the original platelet population. **(c)** Neutrophil in whole blood and in neutrophil-depleted blood are shown. **(d)** Platelet depletion in mice. Before depletion (Time -1) and 1h after injection of isotype control, blue dots (n=3) or platelet depletion antibody, red dots (n=8). Mean ± s.d. **(e)** Area in microns^2 of clots in control mice (n= 4 mice,16 clots) and platelet depleted mice (n=4 mice, 27 clots). Mean ± s.e.m is shown. Student's t test. ****P < 0.0001. **(f)** H&E staining of lung from a platelet depleted mouse treated with HIT IgG plus heparin. Clot is indicated by the arrow. Images were taken with a Zeiss Axioskop microscope running Zen software version 2.3.64.0 with a 40X objective. Scale bar 20 μm. **(g)** Confocal image of a clot in the lung of a platelet depleted mouse treated with HIT IgG plus heparin. Hylite-647-labelled fibrin (red) and DAPI-stained cell nuclei (blue). Scale bar 20 μm. Source data for (d, e) are provided as a Source Data file.

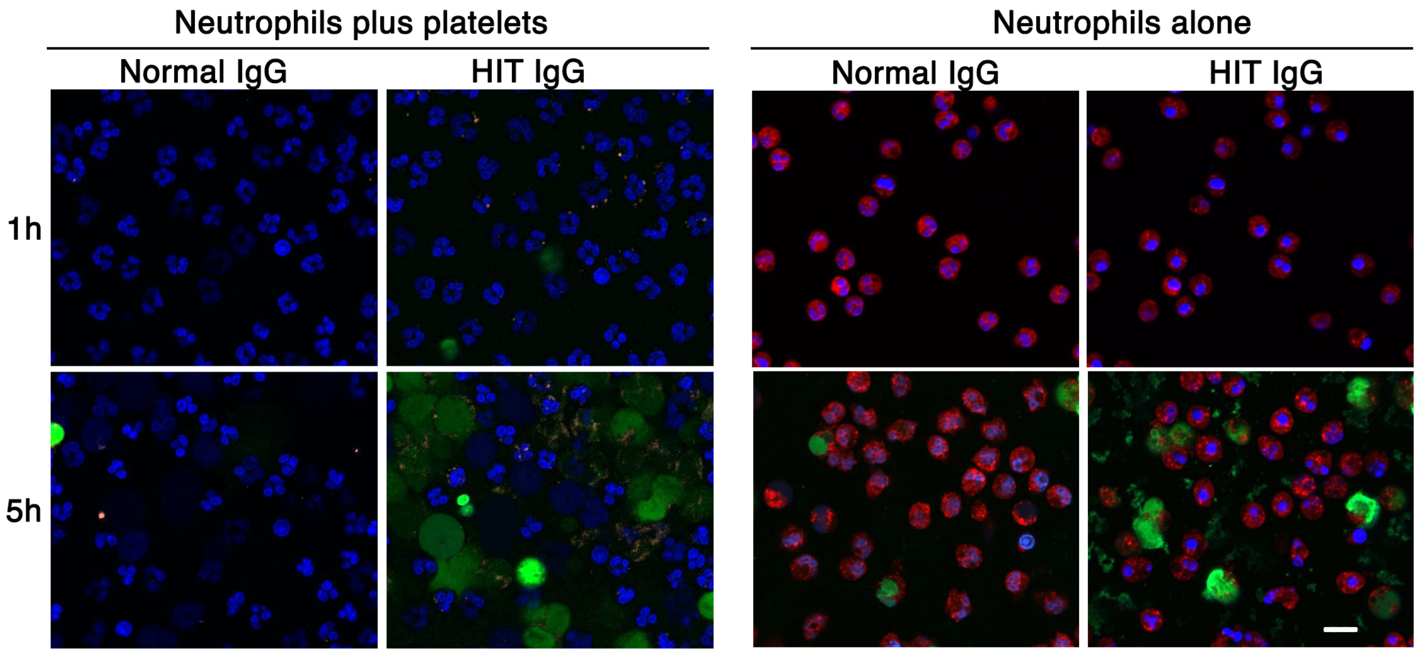

Supplementary Figure 6. Purified neutrophils were incubated at 37°C for 5h with either CM Orange-stained activated platelets (left panels) or in the absence of platelets (right panels). Cells were treated with normal IgG or HIT IgG plus PF4 and heparin in the presence of Sytox green (green). Nuclear DNA stained with Hoechst shown in blue. Neutrophils (red) in right panel were stained with Deep Red. Scale bar, 10 μm.

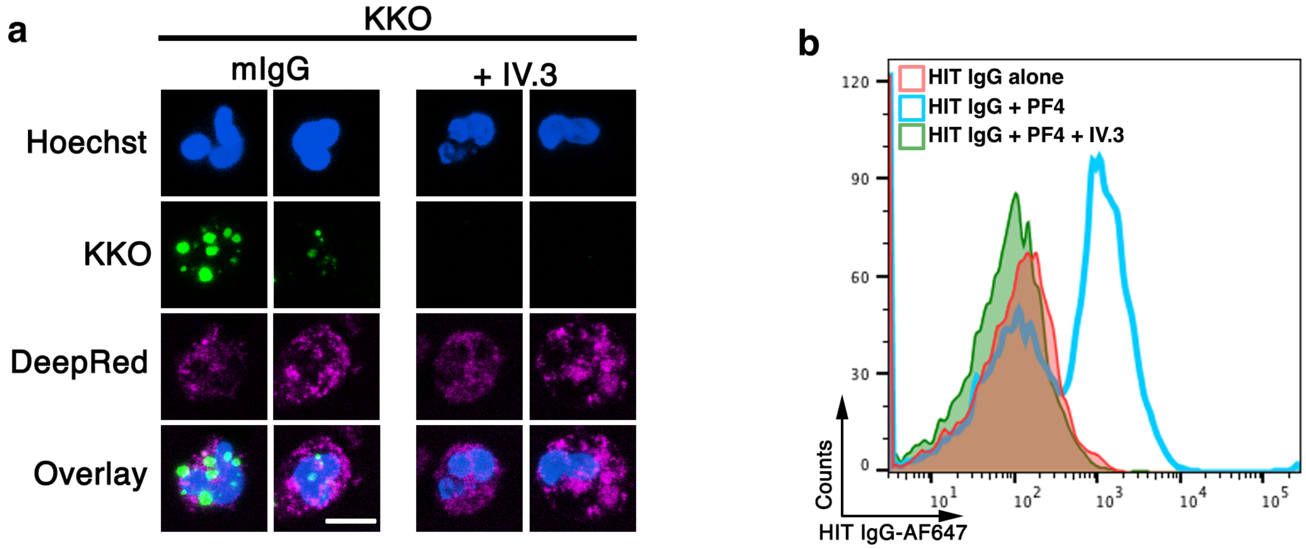

Supplementary Figure 7. (a) Purified neutrophils were stained with Deep Red cell staining and incubated with Alexa 488-labeled KKO plus PF4 and heparin in the presence of normal mouse IgG (left panels) or IV.3 antibody (right panels). Cells were cytospun on slides, fixed, mounted with DAPI mounting media and imaged by confocal microscopy. Scale bar, 5 μm. **(b)** Platelets were incubated with AlexaFluor 647-labelled HIT IgG plus heparin in the absence (red histogram) or presence (blue histogram) of PF4. Addition of IV.3 antibody inhibited binding of HIT IgG to platelets (green histogram).

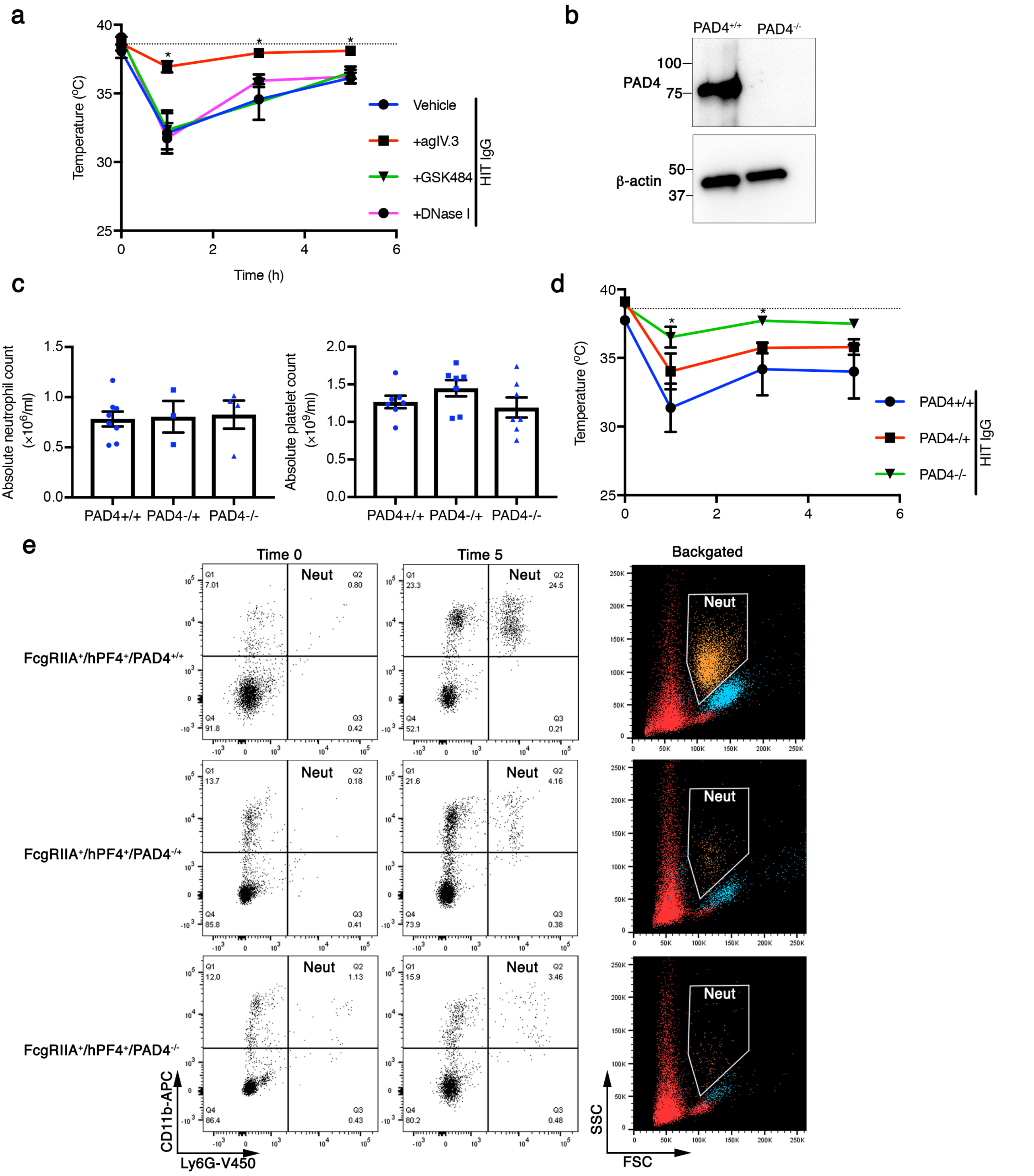

Supplementary Figure 8. (a), Rectal temperature after treatment of *FcγRIIa⁺/hPF4⁺* mice with HIT IgG, or HIT IgG plus Ag. IV.3, GSK484 or DNase I (n=4). Mean ± s.e.m are shown. **(b),** Western blots of PAD4 in bone marrow cells from *PAD4⁺/⁺* and *PAD4⁻/⁻* mice probed with anti-PAD4 antibody. β-actin was used as loading control. **(c),** Neutrophil counts (left panel; P>0.05) and platelet counts (right panels; P>0.05) of *FcγRIIa⁺/hPF4⁺* double transgenic mice wild-type, heterozygous or knockout for PAD4 (*PAD4⁺/⁺*, *PAD4⁻/⁺* or *PAD4⁻/⁻*, respectively). No significant differences were observed. Mean ± s.e.m are shown.
(d), Rectal temperature after treatment of *FcγRIIa⁺/hPF4⁺* double transgenic mice wild-type, heterozygous or knockout for PAD4 (*PAD4⁺/⁺*, *PAD4⁻/⁺* or *PAD4⁻/⁻*, respectively) with HIT IgG plus heparin (n=4 for *PAD4⁺/⁺*, n=5 for *PAD4⁻/⁺* and *PAD4⁻/⁻*). Mean ± s.e.m are shown. Dotted line represents the mean body temperature of mice before treatment (38.6ºC, n=60). Statistical analyses: Kruskal-Wallis test adjusted for multiple comparisons by Holm's stepdown Bonferroni procedure. *P <0.05

(e), Peripheral blood mononuclear cells (PBMC) from *FcγRIIa*$^{+}$*/hPF4*$^{+}$ double transgenic mice wild-type, heterozygous or knockout for *PAD4*; *PAD4*$^{+/+}$, upper panels; *PAD4*$^{-/+}$, middle panels or *PAD4*$^{-/-}$, lower panels; before treatment (Time 0) or after treatment (Time 5h) with HIT IgG plus heparin. Histopaque was used to separate PBMCs. Neutrophils in the PBMC layer (low density granulocytes, LDG) were detected by staining with anti-CD11b and anti-Ly6G. Low density neutrophils are double positive cells in the upper right quadrants. The low density neutrophils (orange) and monocytes (blue) from *PAD4*$^{+/+}$, *PAD4*$^{-/+}$ or *PAD4*$^{-/-}$ transgenic mice were backgated and are shown in the right panels within the total PBMC population (red dots) at Time 5h. Neut, neutrophils. Source data for (a, b, c, d) are provided as a Source Data file.

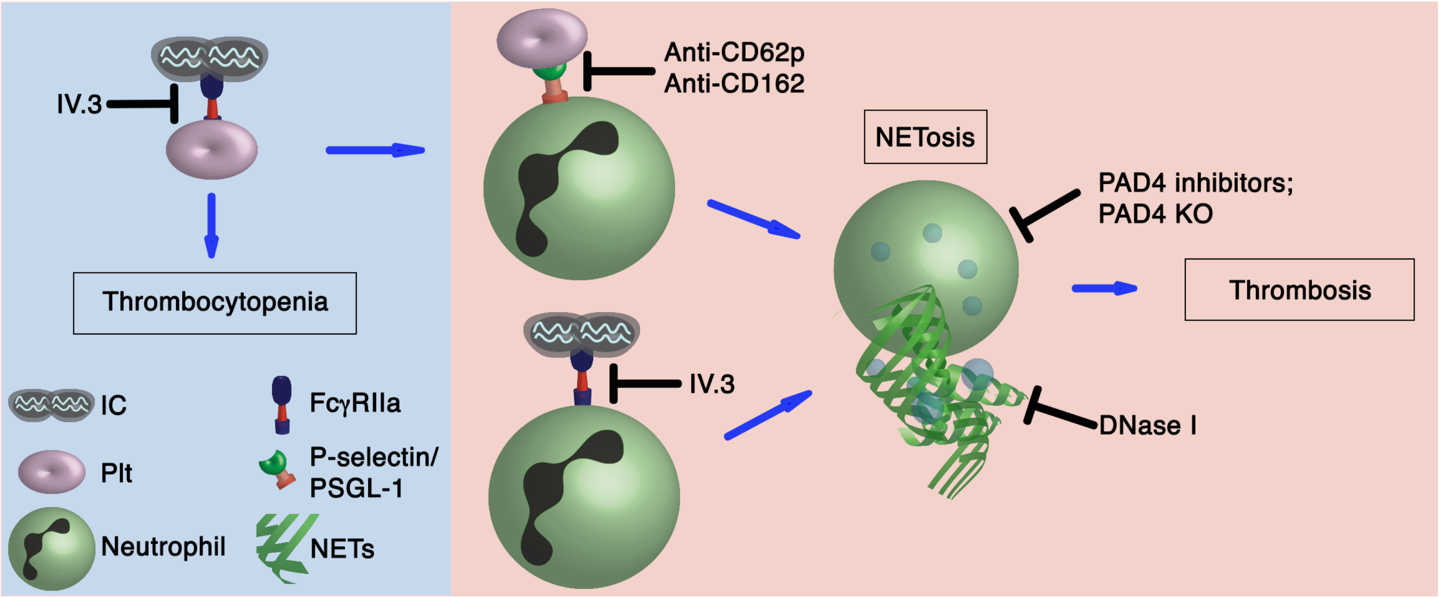

Supplementary Figure 9. Schematic diagram of the effects of HIT immune complexes (ICs). HIT ICs engage FcγRIIa on platelets and neutrophils. For platelets this interaction results in platelet activation, which causes thrombocytopenia. In addition, activated platelets interact with neutrophils via P-selectin/PSGL-1 to form neutrophil-platelet aggregates. For neutrophils, both HIT IC interaction with FcγRIIa or neutrophil-platelet aggregates lead to NETs formation, which is essential for thrombosis in HIT. Both thrombocytopenia and thrombosis can be inhibited by blocking FcγRIIa with the IV.3 antibody, while inhibition of NETs formation or NETs digestion with DNase I stops thrombosis but does not affect thrombocytopenia. Prevention of neutrophil-platelet association with anti-CD62p or anti-CD162 does not block NETosis and thrombosis due to the independent interaction of neutrophils with HIT IC via FcγRIIa. Arrows indicate the direction of the reaction while bar-headed lines denote inhibition. IC, HIT immune complex; Plt, platelet; KO, knockout.
